# Spectroscopic Relationship between XOD and TAOZHI Total Polyphenols Based on Chemometrics and Molecular Docking Techniques

**DOI:** 10.3390/molecules29184288

**Published:** 2024-09-10

**Authors:** Mingyu Yang, Yitang Xu, Qihua Yu, Mengyu Li, Liyong Yang, Ye Yang

**Affiliations:** 1 School of Pharmacy, Guizhou University of Traditional Chinese Medicine, Guiyang 550025, China; yangmingyu5970@163.com (M.Y.); xuyitang4879@163.com (Y.X.); yuqihua7415@163.com (Q.Y.); limengyu411@163.com (M.L.); 2 Guizhou Key Laboratory for Raw Material of Traditional Chinese Medicine, Guizhou University of Traditional Chinese Medicine, Guiyang 550025, China

**Keywords:** *Prunus persica* (L.) Batsch, total polyphenol extraction, xanthine oxidase, chemometrics, spectral effect correlation analysis, ultra-high performance liquid chromatography

## Abstract

Xanthine oxidase (XOD) is a key enzyme that promotes the oxidation of xanthine/hypoxanthine to form uric acid, and the accumulation of uric acid leads to hyperuricaemia. The prevalence of gout caused by hyperuricaemia is increasing year by year. TAOZHI (TZ) can be used for the treatment of rheumatic arthralgia due to qi stagnation and blood stasis and contains a large number of polyphenolic components. The aim of this study was to investigate the relationship between chromatograms and XOD inhibition of 21 batches of TZ total polyphenol extract samples. Chemometric methods such as grey correlation analysis, bivariate correlation analysis, and partial least squares regression were used to identify the active ingredient groups in the total polyphenol extracts of TZ, which were validated using molecular docking techniques. The total polyphenol content contained in the 21 batches did not differ significantly, and all batches showed inhibitory effects on XOD. Spectroeffect correlation analysis showed that the inhibitory effect of TZ on XOD activity was the result of the synergistic effect of multiple components, and the active component groups screened to inhibit XOD were F2 (4-O-Caffeoylquinic acid), F4, and F10 (naringenin). The molecular docking results showed that the binding energies of all nine dockings were lower than −7.5 kcal/mol, and the binding modes included hydrogen bonding, hydrophobic forces, salt bridges, and π-staking, and the small molecules might exert their pharmacological effects by binding to XOD through the residue sites of the amino acids, such as threonine, arginine, and leucine. This study provides some theoretical basis for the development and utilisation of TZ total polyphenols.

## 1. Introduction

Xanthine oxidase (XOD) is a pivotal enzyme that facilitates the oxidation of xanthine/hypoxanthine to uric acid [1]. It plays a crucial role in the biosynthesis of purine to uric acid in various organisms. Hyperuricaemia is a condition characterised by the accumulation of uric acid in the body, exceeding the capacity for excretion [2]. Allopurinol is a pharmaceutical agent that has been granted clinical approval for the treatment of hyperuricaemia [3]. The process of uric acid conversion can be interrupted by the inhibition of XOD activity. However, these drugs are associated with a number of adverse side effects, including allergic and hypersensitivity reactions, cardiovascular disease, and chronic liver disease [4,5]. It is, therefore, imperative to develop natural plant extracts that can inhibit XOD for the treatment of hyperuricaemia.

TZ is the dried branch of the persimmon tree (*Prunus persica* (L.) Batsch), belonging to the genus Prunus and family Rosaceae. It has been demonstrated to have efficacy in the activation of blood circulation and the removal of blood stasis, as well as in the detoxification and killing of insects. It is frequently employed in the management of dysmenorrhoea, cardiac and abdominal discomfort, rheumatic arthralgia, and other ailments resulting from qi stagnation and blood stasis [6,7]. A number of studies have demonstrated that TZ contains a variety of compounds, including polyphenols, flavonoids, alkaloids, organic acids, and volatile oils [8]. Among these, polyphenols and flavonoids have been identified as possessing particularly valuable pharmacological properties and significant potential for exploitation [9,10].

The current body of research on *Prunus persica* (L.) Batsch has concentrated on the metabolomic and transcriptional differences observed in the peach’s leaves, flowers, and fruits [11,12,13]. Additionally, studies have been conducted on the clinical applications of peach kernels in formulations, the identification of polysaccharides in peach gum, and the microbiology of the peach root zone [14,15,16]. With regard to TZ, only a limited number of studies have reported the analysis of its constituent components [8,10], as well as the impact of TZ compost on microorganisms and its efficacy in the treatment of wilt [17]. The extraction of total polyphenols from TZ and the undertaking of fingerprinting studies have yet to be reported in the literature.

Hyperuricemia will lead to rheumatic disease gout in the later stage, and traditional Chinese medicine believes that gout and hyperuricemia are the manifestations of phlegm and blood stasis. At present, the clinical treatment of hyperuricaemia is mostly related to the inhibition of xanthine oxidase, and the commonly used xanthine oxidase inhibitors and drugs include allopurinol, febuxostat, topiostat, etc. [18], and there is still a large space for the research of designing and developing the inhibitors based on the main raw materials of Chinese herbs. TZ has the effect of promoting blood circulation and removing blood stasis and can be used to treat rheumatic joint pain diseases [19]. Studies have shown that polyphenols can improve the symptoms of rheumatic diseases [20]. However, it is unclear whether the polyphenols in TZ have the potential to inhibit XOD activity. Therefore, this study started with the total polyphenol components of TZ (TZ–TPC), determined the difference of total polyphenol content between different batches, analyzed the batch components by UPLC fingerprint, combining with the IC_50_ value of its inhibitory effect on XOD, preliminarily screened out the component group that inhibited XOD in TZ.

The fingerprint of traditional Chinese medicine is an effective method for elucidating the relative relationship between the chemical components present in medicinal materials [21,22]. This method is an effective means of characterising, comprehensively evaluating, and controlling the internal quality of traditional Chinese medicine. Furthermore, chemometrics methods, including grey correlation analysis (GRA), bivariate correlation analysis (BCA), and orthogonal partial least squares discriminant analysis (OPLS-DA) [23,24,25], are cross-applications of computer science and pharmacodynamics to study the spectrum–effect relationships of herbal medicines using multidimensional joint analysis. These analytical techniques are capable of elucidating the intricate interrelationships between multiple factors and variables [23].

Consequently, this study initially examined the methodology for the extraction of total polyphenols from TZ. A total of 21 batches of TZ–TPC were extracted, and their contents were determined. Subsequently, the optimal reaction conditions for XOD were determined, and the inhibitory effect of each batch on XOD was evaluated. UHPLC was employed to establish the batch characteristic fingerprints of TZ. Based on the fingerprint of TZ and its inhibitory effect on XOD, a comprehensive evaluation of the quality characteristics of TZ was conducted. This entails the utilisation of an array of techniques, encompassing spectrum–effect relationship analysis and molecular docking for the screening and verification of the component groups of TZ that inhibit XOD activity. The objective is to establish a more comprehensive and accurate quality evaluation system that can serve as a reference for its future development and utilisation.

## 2. Results

### 2.1. The Optimal Extraction Conditions for TZ-TPC

The standard curves of different concentrations of rutin were obtained according to the method under Section 4.2 (see Figure S1), and the resultant equations were y = 0.0691x + 0.0351 and R^2^ = 0.9994, indicating that the linearity of rutin was good between the concentrations of 0.1 and 0.6 mg/mL. The experiment was performed by the one-way controlled variable method; firstly, the extraction conditions were fixed as extraction time 50 min, material–liquid ratio 1:30 (g/mL), and extraction temperature 50 °C, and then, the five factors in Table 1 were examined sequentially, the absorbance values measured by each method were brought into the standard curve, and the yield of total flavonoids obtained by each method was calculated to judge the optimal extraction conditions.

The results are shown in Table 1, and the optimal extraction conditions of TZ–TPC were obtained as follows: take 1 g of TZ sample and add 40 mL of ethanol with a volume fraction of 60%; ultrasonic extraction was carried out at 50 °C for 80 min and then filtered after cooling to obtain the filtrate for spare parts.

### 2.2. Extraction Rate of Different Batches of TZ–TPC

**Figure 1:**
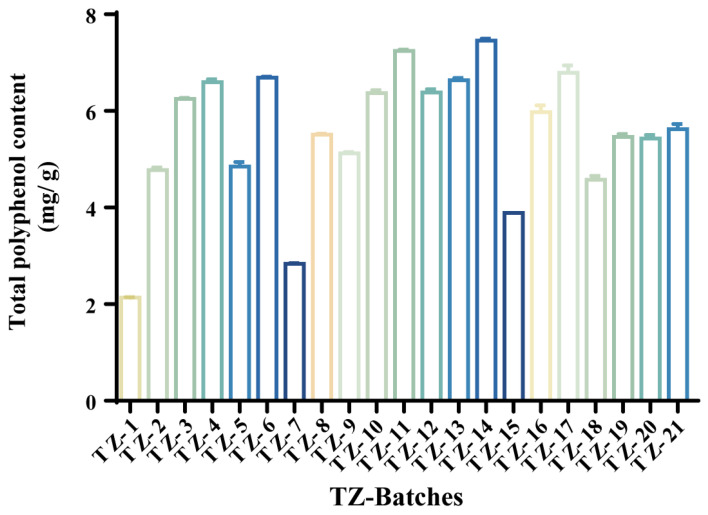
Histogram of total polyphenol yields of batches TZ–1-TZ–21 (*n* = 3).

According to the optimal extraction conditions obtained from the screening, the total polyphenols of 21 batches of TZ samples were extracted according to the method of test preparation under Section 4.2. Their yields were calculated (see Figure 1), a one-way ANOVA was performed to analyse the differences in the content between batches (Table S1), and the total polyphenol yields of each batch were ranked from highest to lowest (Table 2). The results showed that there were more significant differences in the total polyphenol content between batches, with TZ–14 having the highest total polyphenol yield of 7.454 ± 0.040 mg/g and TZ–1 having the lowest yield of 1.440 ± 0.058 mg/g. One-way ANOVA revealed that the differences in total polyphenol content between the TZ–1, –3, –7, –9, –12, –14–16, and –18 batches, and the remainder were more pronounced (*p* < 0.01). Conversely, the total polyphenol content of the latter batches did not diverge significantly. The findings suggest that there is considerable heterogeneity in the total polyphenol content of the samples from disparate origins.

### 2.3. Optimal Reaction Conditions for XOD

**Figure 2:**
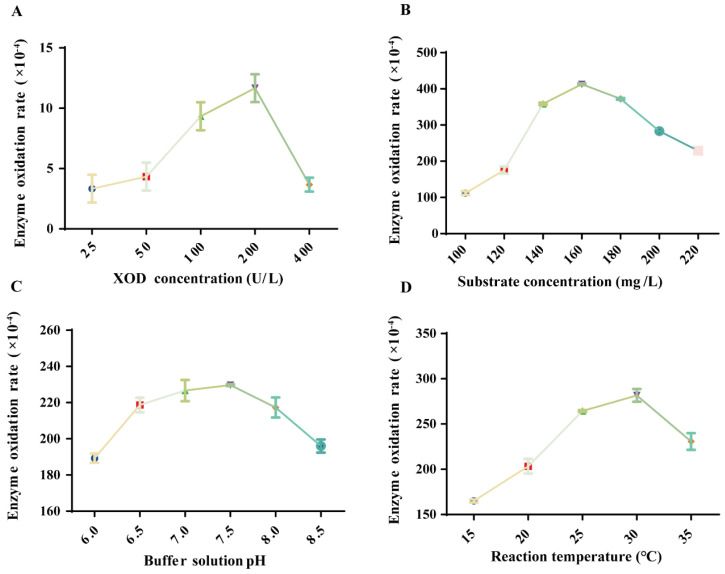
Screening of optimal reaction conditions for XOD (*n* = 3). (**A**) XOD concentration screening. (**B**) Xanthine substrate concentration screening. (**C**) Screening of PBS buffer pH. (**D**) Reaction temperature screening.

As can be seen from Figure 2 and Table 3, the optimal reaction conditions for XOD were screened by one-way experiments as follows: the concentration of XOD in the reaction was 200 U/L, the concentration of xanthine substrate was 160 mg/L, the temperature of the reaction was 30 °C, and the pH of the PBS buffer was 7.5.

### 2.4. The Inhibition of XOD Activity by Each Batch of TZ Was Determined, and the Enzyme Reaction Kinetics Was Judged

**Figure 3:**
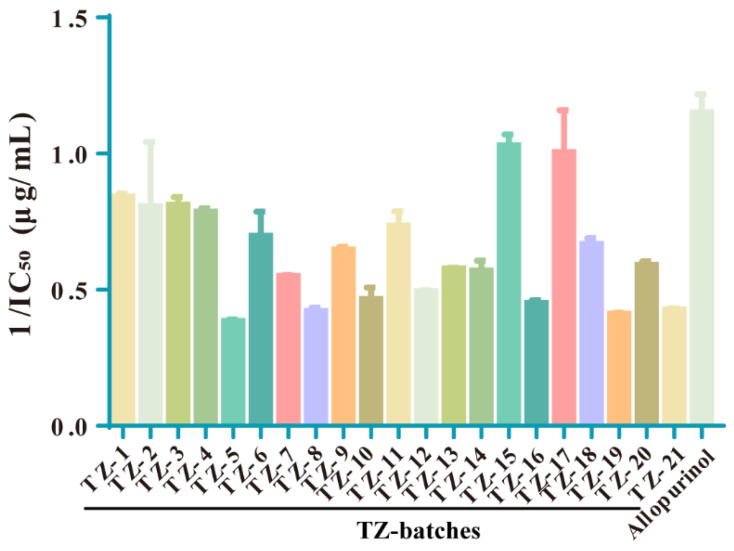
IC_50_ value of XOD activity inhibition by 21 batches with positive drug allopurinol.

The IC_50_ values of XOD inhibition by 21 batches of TZ–TPC were determined according to the screened XOD optimal reaction conditions, and the results are shown in Figure 3 and Table 4, while the IC_50_ values of XOD inhibition by allopurinol were also determined. Graphs were plotted using GraphPad Prism software v6.01, and one-way ANOVA was performed to compare the IC_50_ values of each batch of TZ for inhibition of XOD activity with those of the positive drug, allopurinol, and the results are shown in Table S2. The activity relationship in response to the IC_50_ value for inhibition of XOD activity is negatively correlated, i.e., the smaller the IC_50_ value, the stronger the inhibitory activity. For the convenience of subsequent analyses, the IC_50_ values were expressed by taking the reciprocal, i.e., the larger the value, the stronger the inhibitory activity. The results showed that all TZ batches had some inhibitory effect on XOD, among which TZ–15 and TZ–17 performed better, and there was no significant difference between the inhibitory effect and that of the positive drugs in the univariate analysis.

The kinetics of enzyme inhibition was investigated in the TZ–XOD reaction system by determining the rate of reaction of TZ on XOD at different concentrations of xanthine substrate solution and graphing it as the reciprocal of the rate of enzyme reaction (1/V) versus the reciprocal of the substrate concentration (1/[s]). According to the position of the intersection of the straight line and the vertical axis of the coordinate axis, the type of inhibition of TZ on XOD was determined. The results showed that TZ had a competitive and inhibitory effect on the formation of uric acid [26] because its Km value increased and the Vmax value remained unchanged, indicating that TZ may directly bind to the active site of XOD, thereby competing with xanthine in the catalytic process (Figure S2).

### 2.5. Fingerprinting of TZ Batches and Methodological Validation

#### 2.5.1. Establishment of TZ Characteristic Fingerprint and Similarity Evaluation

The results of precision, stability, and reproducibility of methodological validation of the fingerprint profiles are shown in Table S3 and Figure S3, and the RSD values of each methodology were <3%, indicating that the prepared test samples were stable within 24 h, the instrumental precision was good, and the experimental methodology was reproducible.

**Figure 4:**
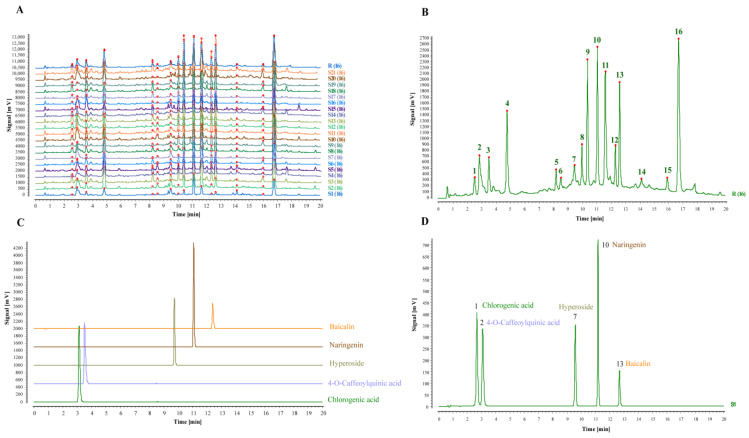
Fingerprint analysis of TZ batches. (**A**) Common patterns in the fingerprint profiles of 21 batches. The different colours in the figure represent the chromatograms of different batches of samples, where the red dots are the common peaks of the marker corrections. (**B**) A total of 16 common peaks were calibrated in the control spectrum obtained in the multi-point correction mode. (**C**) Fingerprint profiles of the five controls. (**D**) Fingerprint profile of the mixed control.

The control R and shared pattern overlay of the fingerprint profiles of 21 batches of TZ–TPC samples were established (Figure 4A). In total, 16 shared fingerprint peaks were calibrated in the profiles of each batch of the samples using the No. 8 peak common to all the samples as the reference peak and its retention time as 1 (Figure 4B), where RPA and RRT were calculated as shown in Tables S4 and S5. By the agreement of retention time and UV absorption spectra of the test solution profile with that of the control (Figure 4C), five common peaks were identified, of which peak 1 was chlorogenic acid, peak 2 was 4–O–Caffeoylquinic acid, peak 7 was hypericin, peak 10 was naringenin, and peak 13 was baicalin. At the same time, the standard was mixed to obtain its fingerprint (Figure 4D).

The similarity of the control profile was set as 1, and the similarity of each sample was calculated according to the software (see Table S6). The similarity of each batch of TZ ranged from 0.901 to 0.993. The similarity between the batches was high, which was greater than 0.9, indicating that the nature of the components was similar between samples from different origins, and the peaks of the components were more stable.

#### 2.5.2. CA and PCA Analyses

**Figure 5:**
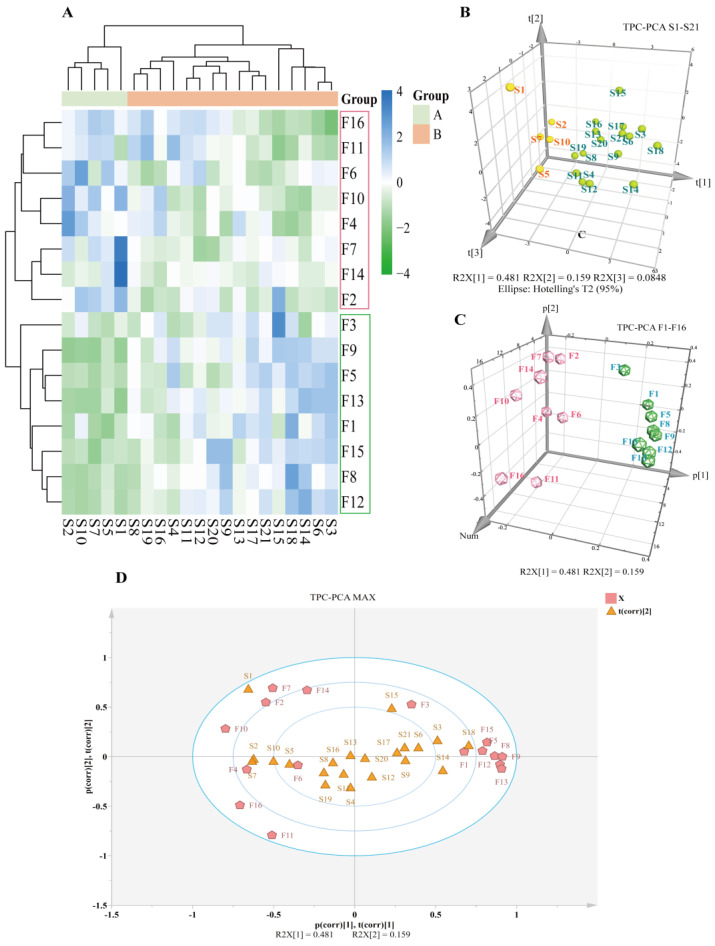
Cluster analysis and principal component analysis of 21 batches of TZ fingerprint profiles. (**A**) Clustering heat map of 21 batches of TZ from different origins. (**B**) Plot of principal component scores for 21 TZ batches. The yellow orb in the figure indicates PCA classification I and the green orb indicates PCA classification II. (**C**) Plot of principal component scores for the 16 shared peaks in the TZ batch. (**D**) Plot of the fraction of batches and shared peaks mixed in PCA (triangles represent batches; pentagrams represent shared peaks). In the figure, S1–21 represents TZ1–21.

The 16 common peak areas identified in the fingerprint profiles of the 21 batches of TZ samples were used as the raw data to construct a 20 × 19 order data matrix, which was subsequently imported into the Microbiology plaTPCorm http://www.bioinformatics.com.cn (last accessed on 30 Aug 2024). The Euclidean distance method was used to generate the clustering heat map, with the coloured intensities ranging from green to blue, representing the colour block content from low to high. The results are shown in Figure 5A. The results of the cluster analysis demonstrated that the 21 batches of TZ were classified into two categories. Batches S1, 2, 5, 7, and 10 were grouped together in Category I, while the remaining batches were placed in Category II. This indicated that the 21 batches of TZ samples exhibited differences in their fingerprint profiles.

A principal component analysis (PCA) was conducted on 21 batches of TZ samples using the multivariate statistical software SIMCA 14.1 in order to calculate their principal component scores (Figure 5B). The two principal components with the largest contribution were identified and excluded from further consideration. The cumulative variance contribution of the first principal component (t {1}) was 48.1%, while the cumulative variance contribution of the second principal component (t {2}) was 15.9%. The total contribution of these two components was 64%, indicating that they could effectively reflect the fundamental characteristics of TZ herbs. The results demonstrated that 21 batches of TZ samples were classified into two distinct classes when the principal component t {1} scores were classified as −2. The outcomes of the PCA scores were found to be in alignment with those of the cluster heat map analysis.

In the clustering analysis of the 16 shared peaks using CA and PCA, the results showed that F, 2, 4, 6, 7, 10, 11, 14, and 16 (pink coils) were clustered into one category, and F1, 3, 5, 8, 9, 12, 13, and 15 (greens coils) were clustered into one category, and the clustering results were consistent between the two (Figure 5C), which indicated that the shared peaks were more consistently categorised in the two analytical methods. A mixed score plot of batch and shared peaks was also demonstrated in PCA (Figure 5D).

### 2.6. Chemometric Analyses

#### 2.6.1. Grey Relevance Analysis (GRA)

The data matrix of GRA was formed by using the inverse of the IC_50_ value measured for inhibition of XOD activity as the reference series and the peak areas of the 16 common peaks as the comparison series. The calculated correlation coefficients of the common peaks of each batch were ranked in accordance with the magnitude of the correlation (see Table 5). A higher ranking indicated a greater contribution of the common peaks to the inhibition of XOD activity. The results showed that the correlations between the 16 common peaks and the results of XOD inhibition activity were all greater than 2, and the correlations between the batches and the pharmacodynamic indexes had little variation, indicating that there was a high degree of correlation between the 16 common peaks and the two variables of XOD inhibition activity.

#### 2.6.2. Bivariate Correlation Analysis (BCA)

**Figure 6:**
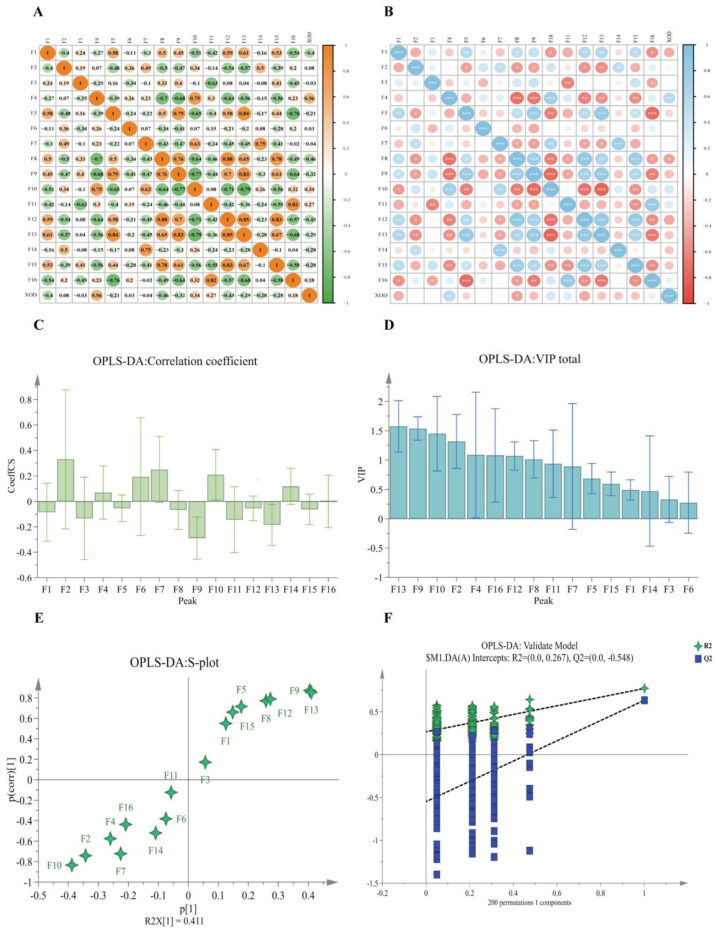
BCA and OPLS–DA results. (**A**) Pearson correlation coefficient plot in BCA (the graph from yellow to green indicates high to low scores). (**B**) *p*-value plot of significance in BCA (the graph from blue to red indicates high to low scores). (**C**) The magnitude of VIP values of shared peaks in OPLS–DA. (**D**) Magnitude of standardised regression coefficients for OPLS–DA common peaks. (**E**) The S–plot in OPLS–DA indicates the degree of data discretization. (**F**) Orthogonal calibration model in OPLS–DA with the number of calibrations set to 200. * Significant at the 0.05 level (two–tailed). ** Significant correlation at the 0.01 level (two–tailed). *** Significant correlation at the 0.001 level (two–tailed).

The inverse Z–score of the IC_50_ value of TZ inhibition of XOD activity was standardised and processed, then imported into SPSS 26.1 software with it as the variable (Y) and the area of each shared peak as the variable (X) to carry out a bivariate correlation analysis and to calculate Pearson correlation coefficients of the area of the shared peaks with the inhibitory activity of XOD and their *p*-values. The results showed that peaks 2, 4, 6, 10, 11, and 16 were positively correlated with TZ inhibition of XOD activity, of which F4 was a significant positive correlation (*p* < 0.05); the rest of the peaks were negatively correlated with TZ inhibition of XOD activity, of which F8 was a significant negative correlation (*p* < 0.05). Significant differences between the shared peaks and the pharmacodynamic indicators were determined in a *p*-value analysis, which showed that only F4 and F8 were significant for the pharmacodynamic indicators (*p* < 0.05), as shown in Table 6 and Figure 6A,B. See Tables S7 and S8 for raw data from BCA.

#### 2.6.3. Orthogonal Partial Least Squares Discriminant Analysis (OPLS-DA)

The area of the 16 common peaks was used as the independent variable, and the inverse results of the IC_50_ values of XOD activity inhibition by TZ–TPC from different batches of the test material were used as the dependent variable and imported into the SMICA14.1 software for OPLS–DA analysis (the data were all standardised by Z–score first). Standardised regression coefficients and variable importance in projection (VIP) values were calculated to evaluate the spectral efficacy relationship of TZ–TPC in inhibiting XOD activity.

In OPLS–DA, positive or negative regression coefficients indicate that the TZ components are positively or negatively correlated with the inhibitory enzyme activity, and the larger the regression coefficient, the greater the contribution to the pharmacodynamic effect of XOD inhibition [27]. The results demonstrated that the total of peaks F2, F4, F6, F7, F10, and F14 exhibited a positive correlation in the inhibition of enzyme activity, with a contribution size of F2 > F7 > F10 > F6 > F14 > F4. Conversely, the remaining peaks exhibited a negative correlation, with a contribution size of F9 > F13 > F9 > F11 > F3 > F1 > F8 > F15 > F12 > F5 > F16 (see Figure 6C and Table S9). It is suggested that the corresponding components in the positive correlation are the material basis for the inhibition of XOD activity and are the active components in which TZ exerts its anti–XOD ability, and this result is basically consistent with the results of the bivariate correlation analysis.

The VIP value indicates the degree of contribution of the component to the activity. In general, a VIP value greater than 1 indicates a significant contribution to the model. It can also be used to describe the degree of explanation of the independent variable to the dependent variable. The larger the VIP value, the greater the ability of the independent variable to explain the dependent variable [28]. In the present study, OPLS–DA–VIP values were analysed for the ability of TZ–TPC to inhibit XOD as a pharmacodynamic index in order to identify the peaks that had the greatest effect on the ability of TZ–TPC to inhibit XOD. This was corroborated by Figure 6D, which demonstrated that the inhibitory effect of TZ–TPC extract on XOD was, in descending order, F13 > F9 > F10 > F2 > F4 > F16 > F12 > F8. It is proposed that the aforementioned components exert a considerable influence on the inhibition of XOD (VIP > 1).

Based on the VIP values and standard regression coefficients, analysing the regression coefficients of the pharmacodynamic indexes under the condition that the VIP value was greater than 1, it was found that there was a positive correlation between peaks 2, 4, and 10 and a negative correlation between peaks 8, 9, 12, 13, and 16 in the process of inhibition of XOD activity by TZ–TPC (see Figure S4). In the meantime, the S–plot in OPLS-DA shows a good degree of data dispersion (Figure 6E,F), while the test model is set to 200, the results show that the absolute value of the model Q2 is greater than 0.5, and the model is highly credible [21].

**Figure 7:**
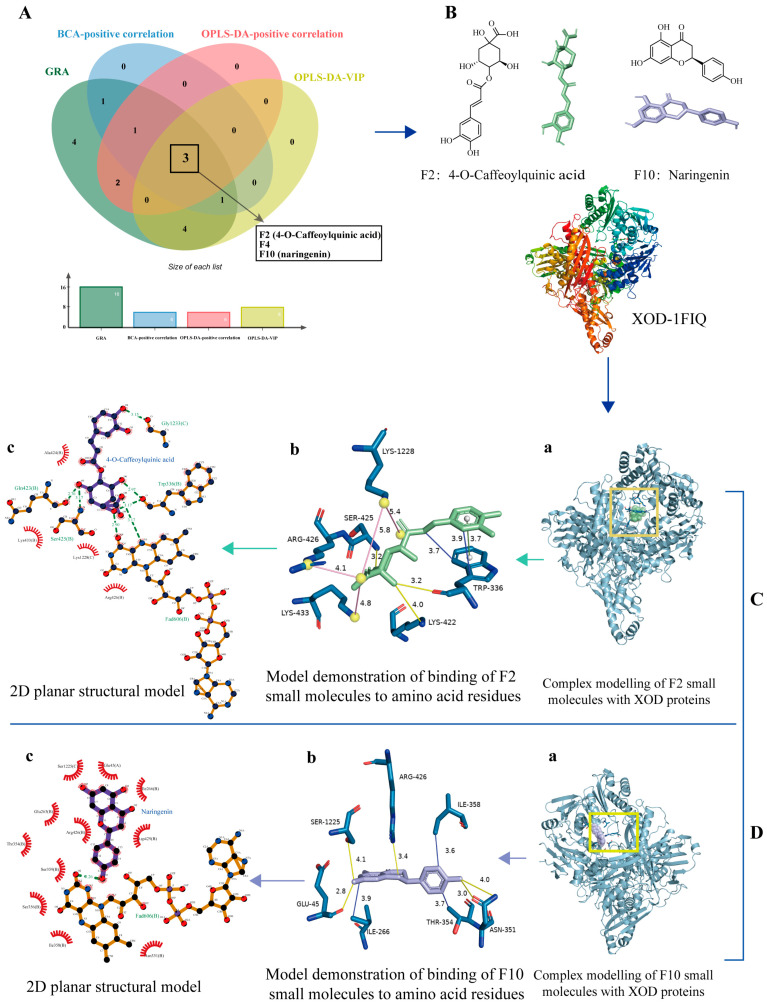
Visualisation of molecular docking results (The arrows in the figure indicate the experimental docking sequence). (**A**) Venn intersection plot for spectral effect correlation analysis. (**B**) 3D structure of two small molecules with XOD proteins. (**C**) Complex conformation of F2 with XOD protein. (**a**) is the overall composite view of F2 binding to the protein, (**b**) is the 3D site plan of F2 binding to amino acid residues of the protein (where the dark blue stick structures are amino acid residues), and (**c**) is the 2D plan view of F2 binding to amino acid residues in the conformation. (**D**) Complex conformation of F10 with XOD protein. (**a**) is the overall composite view of F10 binding to the protein, (**b**) is the 3D site plan of F10 binding to amino acid residues of the protein, and (**c**) is the 2D plan view of F2 binding to amino acid residues in the conformation.

Based on the conclusions of the three spectrum–effect analysis methods, we selected the peaks with high correlation and positive correlation in BCA and OPLS–DA to inhibit XOD activity and VIP value > 1 for further molecular docking semi-flexible docking verification. The results are shown in Figure 7A.

### 2.7. Molecular Docking of Constituent Groups to Proteins

After taking the intersection of the three spectral analysis methods in Venn, three core components were obtained, which were F2, F4, and F10, among which F2 was identified as 4–O–Caffeoylquinic acid, and F10 was identified as naringenin, so both of them were selected for the molecular docking validation. The 3D structures of 4–O–Caffeoylquinic acid (PubChem CID: 9798666) and naringenin (PubChem CID: 439246) were downloaded from the PubChem (https://pubchem.ncbi.nlm.nih.gov/) (accessed on 2 September 2024) website (Figure 7B). XOD water molecules and their original ligands were removed using PyMOL [29].

Hydrogenation and charge calculations were then performed using Autodock 4.2.6 and saved in PDBQT format for backup. The coordinates of the centre of the docking box of the protein with the small molecule 4–O–Caffeoylquinic acid were x = 28.673, y = 29.925, and z = 101.421. The coordinates of the centre of the docking box of the protein with the small molecule naringenin were x = 28.231, y = 29.925, and z = 101.421. The two small molecules were Autodock vina docked to the XOD proteins nine times each, and the binding energies obtained from docking both were less than −7 kcal/mol, with F2 binding to XOD in the lowest conformation with a binding energy of −8.2 kcal/mol and F10 binding to XOD in the lowest conformation with a binding energy of −8.6 kcal/mol (Table S10).

The docking results were imported into PyMOL and saved in PDB format for the complexes formed with the large proteins. Subsequently, the results were analysed using the PLIP website, and as can be seen in Table 7, there are a total of four interaction forces in the binding model, including hydrophobic forces, hydrogen bonding, salt bridges, and pi-stacking, which suggests that the binding conformation is stable while the residues linked to the small molecule involve lysine, tryptophan, serine, arginase, isoleucine, threonine, and glutamine, to name a few.

The 3D results were visualised and rendered in PyMol (Figure 7C,D), after which the 2D results were validated using the Liplot+ v.2.2 software (Figure 7C(c),D(c)). Figure 7C(a),D(a) are the binding complex conformations of XOD protein structure and small molecules, where C–a green is F2, D–a purple is F10, and small molecules are spheres model. Figure 7C(b),D(b) are the conformation and interaction force of the binding of small molecules to protein amino acid residues. The yellow solid line represents the hydrogen bonding force, the blue solid line represents the hydrophobic force, the red solid line represents pi-stacking, and the pink solid line represents the salt bridge. Figure 7C(c),D(c) is the 2D conformation of small molecules binding to protein amino acid residues. In the drawing of the 2D plane, the green dotted line represents the hydrogen bond force, and the result is slightly different from the result in PLIP. This discrepancy may be attributed to the inconsistent algorithms employed in the molecular docking results across different software platforms. Nevertheless, both approaches demonstrate that the docking model exhibits favourable scores and a stable binding. The amino acid residues combined with small molecules and their specific binding sites, as well as the magnitude of the interaction between the two, are detailed in Table 7.

## 3. Discussion

### 3.1. Determination of Total Polyphenol Content and XOD Activity of the Batch

Many studies have shown that TZ contains a large number of polyphenols. In this study, we investigated the optimal extraction conditions for its total polyphenols and determined the total polyphenol content in 21 batches of TZ. The top five batches of samples with the highest content were TZ–14, –11, –17, –6, and –13 (from highest to lowest). In addition, the results showed very little variation in content between batches, suggesting that the nature of the total polyphenol content of TZ is relatively stable among several different origins.

XOD catalyses the production of uric acid from xanthine and hypoxanthine [30]. In clinical practice, effective drugs for the treatment of XOD–induced hyperuricaemia often produce side effects that seriously affect the prognosis of patients, which has led to an increasing interest in the extraction of anti–XOD-active components from plants and natural products [31]. Under different conditions, the reaction rate of enzyme and substrate is different; therefore, in this study, the optimal reaction conditions for XOD were first determined, and the type of inhibition of XOD by TZ–TPC was designated as competitive inhibition. Subsequently, the magnitude of IC_50_ values of 21 batches of TZ–TPC inhibiting XOD activity was determined. The results showed that the top five batches with the strongest inhibitory ability were TZ–15, –17, –1, –4, and –3, among which the inhibitory activity of batches TZ–15 and –17 was closest to that of the positive drug allopurinol. It is noteworthy that TZ–15 and TZ–1 demonstrated remarkable efficacy in XOD inhibitory activity yet exhibited relatively low levels of total polyphenol content. This observation suggests that the active ingredients in this batch of herbs, aside from the total polyphenol components, may possess inhibitory properties against XOD. TZ–17 demonstrated remarkable efficacy in both content determination and the inhibition of XOD activity, indicating a positive correlation between polyphenols and XOD activity inhibition in this particular batch.

### 3.2. Batch UHPLC Fingerprints

In order to gain greater insight into the compositional differences between batches, the samples were extracted under conditions that optimised the total polyphenol extraction. This allowed the establishment of batch fingerprints for use in similarity, CA and PCA. The UHPLC superimposed feature profiles demonstrated that the chemical compositions of the TZ from disparate origins exhibited a general similarity, albeit with slight variations in the content of the components. Furthermore, the fingerprint profiles of each batch of herbs demonstrated a high degree of similarity, with 16 common peaks being identified; F1 was chlorogenic acid, F2 was 4-O-Caffeoylquinic acid, F7 was hypericin, F10 was naringenin, and F13 was baicalin. Furthermore, the outcomes of the CA and PCA analyses indicated that samples TZ–1, –2, –5, –7, and –10 were categorised as Class I, whereas TZ–3, –4, –6, –8, and –9, along with samples 11 through 21, were classified as Class II. In comparison to the Class I samples, the Class II samples exhibited a relatively larger peak area in the fingerprint superposition mode, a higher colour block content in the heat map analysis, and a higher PCA score. The discrepancies observed may be attributed to the disparate geographical origins of the batches under consideration. Even when regionally similar batches are subjected to cluster analysis, they do not necessarily display identical behavioural patterns. To illustrate, the samples comprising batch TZ–1–7 were all sourced from Dangwu Town, situated within Guiyang City, Guizhou Province. The results of the clustering and scoring process indicate that TZ–3, TZ–4, and TZ–6 are classified as belonging to Class II, while the remaining samples are classified as belonging to Class I. Similarly, TZ–8 and TZ–10 are from Wangmo County, Xingyi City, Guizhou Province. However, TZ–8 is classified as belonging to Class II, while TZ–10 is classified as belonging to Class I.

### 3.3. Spectral Effect Correlation Analysis (SECA)

To better determine and analyse the pharmacodynamic effects of the ingredients contained in each batch on XOD, the results of the ‘spectra’ and ‘effects’ were fitted using spectral analysis. In the present study, 16 shared peaks in the TZ fingerprint profile were identified as exhibiting a high degree of correlation with XOD inhibitory activity, with correlation coefficients exceeding 0.2, using the GRA method. This indicates that TZ exerts its XOD inhibitory activity as a result of the synergistic effect of multiple components. To gain a more comprehensive understanding of the spectral-pharmacodynamic relationship, we also performed BCA and OPLS–DA analyses of the 16 shared peaks in the TZ fingerprints in combination with XOD pharmacodynamic indices. Pearson’s correlation coefficient in BCA demonstrated that F4 exhibited a markedly positive correlation with the inhibition of the pharmacodynamic activity of XOD, whereas F8 displayed a markedly negative correlation. Furthermore, the *p*-value analysis revealed that F4 and F8 exhibited a statistically significant correlation, indicating that F4 may play a pivotal role in enhancing the inhibitory effect of TZ on XOD activity, while F8 may exert an inhibitory effect. In the OPLS–DA analysis, components with a VIP value greater than 1 were identified, and the correlation coefficients were combined to determine the contribution of each peak to the pharmacodynamic index. The results demonstrated a positive correlation between peaks F2, 4, and 10 and a negative correlation between peaks 8, 9, 12, 13, and 16 in terms of XOD inhibition, indicating that the three peaks F2, 4, and 10 exert a significant influence on the inhibitory activity of XOD.

### 3.4. Molecular Docking Analysis

Combining the three spectral correlation methods, F2 (4–O–Caffeoylquinic acid), F4, and F10 (naringenin) were identified as positively correlated peaks significantly associated with the inhibition of XOD activity; among them, 4–O–Caffeoylquinic acid and naringin have been shown to have strong antioxidant activity [32,33,34]. Higher levels of these peaks indicate stronger inhibition of XOD activity by TZ. The inhibitory effect of TZ on XOD activity was further verified by BCA and OPLS–DA, which demonstrated that this effect was the result of the synergistic effect of multiple compounds within TZ. In the meantime, the results demonstrated that not only components that enhance the inhibition of XOD activity but also components that attenuate the inhibition of XOD activity are present in TZ. Subsequently, the binding between the screened F2, F10, and XOD was verified using the molecular docking technique. The results demonstrated that the molecular binding energy scores of the binding conformations were low and that the interactions were more abundant and strong, indicating that the binding models exhibited good affinity and stability. Furthermore, the results indicate that F2 and F10 may inhibit XOD activity by binding to specific residue sites, including arginine, tryptophan, glutamic acid, serine, and threonine. The binding modes of F2 are more diverse than those of F10, including salt bridging and pi-stacking. Additionally, F2 has a greater number of amino acid residues, which may indicate that it binds to XOD in a more stable manner and has a stronger inhibitory effect.

Currently, there are few studies on the identification of chemical components of TZ. In subsequent experiments, we will consider combining mass spectrometry techniques to identify the components of shared peaks that have not yet been clarified in the fingerprints. Meanwhile, we will also combine the affinity ultrafiltration–ligand capture technique to obtain the XOD-bound components of TZ more accurately. We will explore the effect of TZ on the in vitro and in vivo activities of XOD and study in depth the expression of genes related to hyperuricaemia involved with XOD.

## 4. Materials and Methods

### 4.1. Reagents and Materials

In total, 21 batches of TZ were collected from different natural growth areas. Sample origins and batch numbers are shown in Table S6. All samples were identified by Associate Professor Yang Ye of the Laboratory of Traditional Chinese Medicine and Pharmacognosy Laboratory, Guizhou University of Traditional Chinese Medicine, as dried branches of *Prunus persica* (L.) Batsch, a plant of the genus Prunus, family Rosaceae.

Xanthine oxidase (X8020), xanthine (110F044, purity ≥ 98%), and allopurinol (SA5630, HPLC ≥ 98%) were purchased from Beijing Solabao Biotechnology Co. (Beijing, China); sodium carbonate anhydrous (CAS:497–19–8) was purchased from Tianjin Yongda Chemical Reagent Co. (Tianjing, China); folinol reagent (CAS:NONE6060) was purchased from Shanghai McLean Biochemical Technology Co. (Shanghai, China); sodium hydroxide (CAS:1310–73–2) was purchased from Chengdu Jinshan Chemical Reagent Co. (Chengdu, China); anhydrous ethanol (XK13–011–14001, AR) was purchased from Tianjin Fuyu Fine Chemical Co. (Tianjin, China); gallic acid (CAS:149–91–7) was purchased from Shanghai Ronghe Pharmaceutical Technology Development Co. (Shanghai, China); sodium chloride (CAS:7647–14–5) was purchased from Chongqing Chuandong Chemical (Group) Co. (Chongqing, China).

Reference standards of Chlorogenic acid, 4–O–Caffeoylquinic acid, hypericin, naringenin, and baicalin (purity ≥98%) were purchased from Chengdu Alfa Biotechnology Co., Ltd. (Chengdu, China); formic acid (K2215763) was obtained from Aladdin Bio-Chem Technology Co., Ltd. (Shanghai, China); chromatographic-grade methanol and acetonitrile were provided from TEDIA. All other chemical reagents were of analytical grade.

### 4.2. Solutions Preparation

Preparation of test and control solutions: one gram of TZ powder was accurately weighed and placed in a 150 mL conical flask. Subsequently, 40 mL of a 60% ethanol solution was added and extracted by ultrasonication (60 W, 49 kHz) at 50 °C for 80 min. The resulting solution was then filtered to obtain the test solution. A 1 mg sample of gallic acid was weighed into a 10 mL volumetric flask, and distilled water was added. The solution was then subjected to ultrasonic waves to obtain a 0.1 mg/mL gallic acid control solution.

Colour Developer Preparation: weigh accurately 4 g of anhydrous sodium carbonate powder into a 20 mL volumetric flask. Add an appropriate volume of distilled water, sonicate until the solid is dissolved, then make up the volume with distilled water and shake well to obtain a 20% sodium carbonate solution (ready for use). The folinol reagent is ready for use.

XOD reaction test solution preparation: take 5.0 g of NaOH powder and put it in a 100 mL volumetric flask to obtain 5% NaOH solution; precision suck 13.70 mL of HCl solution with a concentration of 36.5%, put it in a 100 mL volumetric flask, and add distilled water to the scale to obtain 5% HCl solution; 1.8 mg of xanthine powder was measured and placed in a 10 mL volumetric flask with distilled water to obtain 180 mg/L xanthine solution configuration; pipette 16 μL of XOD stock solution (6250 U/L) in a 1 mL volumetric flask and add PBS solution to fix the volume to the scale line (the whole process of configuration was in an ice–water bath); that is, the 100 U/L XOD solution was obtained. Weigh 4 mg of allopurinol in a 10 mL volumetric flask and then fix the volume with 60% ethanol to obtain 0.400 mg/mL allopurinol mother liquor.

### 4.3. Colour Development Method

Refer to the method and modify [35]. A total of 50 μL of the test solution was transferred into a 20 mL brown volumetric flask. Subsequently, 1 mL of folinol reagent and 2 mL of 20% sodium carbonate solution were added dropwise, and the reaction was allowed to proceed for a period of 3 min. Thereafter, the solution was diluted with distilled water. The reaction was then allowed to stand for a further 30 min, after which the absorbance was measured at 760 nm.

### 4.4. Plotting of Standard Curve and Determination of TZ–TPC Content

Take 0.2, 0.3, 0.4, 0.5, and 0.6 mL of the gallic acid standard solution, as described in Section 4.2, and transfer to a 10 mL volumetric flask. The colour should be developed in accordance with the methodology outlined in Section 4.3, after which the absorbance value should be measured. The standard curve is generated by linear regression, with absorbance as the vertical coordinate and mass concentration as the horizontal coordinate.

Take 50 μL of TZ test solution from solution Section 4.2 and transfer it to a 20 mL volumetric flask. The colour should be developed in accordance with the methodology outlined in Section 4.3, after which the absorbance value should be measured. This value should then be substituted into the standard curve in order to calculate the concentration of polyphenol compounds present in the sample. Finally, the total polyphenol yield of TZ can be calculated using the following formula:

$$Total\;polyphenol\;yield\;(mg/g)=\frac{C\times N\times V}{W}$$

where *W* is the TZ weighing volume (g); *N* is the dilution of TZ extract; *C* is the total polyphenol concentration (mg/mL); *V* is the sampling volume (mL).

### 4.5. Optimisation of Total Polyphenol Extraction Conditions and Determination of TPC in 21 TZ Batches

The fixed conditions for the initial one-way investigation of TZ–TPC extraction were as follows: ethanol concentration of 50%, volume of 25 mL, extraction temperature of 50 °C, ultrasonic extraction time of 50 min, ultrasonic frequency of 49 KHz, and ultrasonic power of 60 W. The selected extraction methods were ultrasonic and reflux; the extraction solvents were methanol and ethanol; the solvent concentration was 40–80%; and the material–liquid ratio was 1:30–1:50 g/mL; the extraction time was 50–90 min, which was used as a one-way experiment to design the influencing factors for the controlled variable method. The TZ sample solution (TZ–1) was prepared accurately according to the sample solution preparation method under ‘Section 4.2’, the polyphenols content was determined according to the method under ‘Section 4.3 and Section 4.4’, and the optimal extraction conditions were selected based on the total polyphenol content extracted.

After screening the optimal extraction conditions, 21 batches of TZ–TPC samples were prepared according to the conditions, and the differences in polyphenol content between the batches were calculated and compared.

### 4.6. Screening of Optimal Reaction Conditions for XOD

In references [31,36,37], the XOD activity condition screening system was set up as follows: 400 μL of PBS solution was added to a 1.5 mL centrifuge tube, followed by the addition of 80 μL of XOD solution. The contents were then shaken well and incubated at a constant temperature of 25 °C for 15 min. Subsequently, 320 μL of xanthine solution was added, and the mixture was shaken well once more. The assay was conducted at 290 nm every 50 s for a total of six times. The impact of varying XOD concentrations (25–400 U/L), substrate concentrations (100–220 mg/L), pH levels of PBS solutions (6.0–8.5), and incubation temperatures (15–35 °C) on XOD activity was examined by maintaining a consistent volume of solvent, incubation time, and assay time.

### 4.7. A Study of the Type of Inhibition of XOD

The method of reference [31] was modified as follows: the fixed reaction volume was maintained, the enzyme concentration was 200 U/L, the incubation time was 15 min, the pH was 7.5, and the temperature was 30 degrees Celsius. A volume of 80 μL of TZ (TZ–1) extract at concentrations of 0.090, 0.018, 0.036, and 0.049 mg/mL was taken, and 320 μL of substrate xanthine solution at concentrations of 12.5, 25, 37.5, and 50 mg/L was added. Subsequently, 320 μL of PBS buffer was added, and finally, 80 μL of XOD. Using the inverse of the reaction rate of the enzyme as the vertical coordinate and the inverse of the concentration of the substrate xanthine as the horizontal coordinate, the Lineweaver–Burk double inverse method was used to determine the type of inhibition of XOD by the herbs of the peach branch.

### 4.8. Determination of XOD Inhibitory Activity of TZ-TPC Batch

In total, 0.7, 0.8, 0.9, 1.0, and 1.1 mL of each batch of TZ-TPC extract was taken in a 5 mL volumetric flask, respectively, and was fixed and set aside. Referring to the literature [36], the experiment was divided into four treatment groups, and the samples were spiked according to Table 8. PBS, XOD solution, and different mass concentrations of TZ extracts were added sequentially to the reaction system, and after the addition of the samples was completed, the samples were incubated in a metal bath for 15 min, and then xanthine solution was added and mixed homogeneously.

The reaction was timed from the addition of xanthine solution for 5 min, and the absorbance A was measured at 290 nm to calculate the inhibition rate. Take the allopurinol original solution under Section 4.2, dilute it to 0.008, 0.016, 0.032, 0.064, and 0.128 mg/mL, add samples according to Table 8, replace the TZ extract with allopurinol solution, and perform tests, measurements, and calculations according to the above steps.

The calculation formula was as follows:

$$inhibition\;rate=(1-\frac{A_{1}-A_{2}}{A_{3}-A_{4}})\times 100\%$$

where *A*_1_–*A*_2_ is the sample group, and *A*_3_–*A*_4_ is the blank control group. The raw drug concentration of TZ extract was plotted against the inhibition rate, and the raw drug concentration corresponding to 50% inhibition was calculated as the semi–inhibitory concentration (IC_50_) using GraphPad Prism 6.01 non-linear fitting.

### 4.9. UHPLC Fingerprinting of 21 Batches of TZ-TPC Extracts

#### 4.9.1. Chromatographic Conditions

UHPLC analyses were carried out on an Agilent Ultra-High Performance Liquid Chromatograph (Agilent Technologies Inc., Santa Clara, CA, USA) equipped with a binary solvent delivery pump, an autosampler, and a diode array detector. Samples were drawn and passed through a 0.22 μm microporous filter membrane. Subsequently, the injection volume of the UHPLC system was set at 2 μL, and the separation was performed on an Agilent Eclipse Plus C18 RRHD (P.N. 959757–902, 2.1 × 50 mm, 1.8 μm) column with the separation temperature set at 25 °C, the flow rate set at 0.2 mL/min, and the detection wavelength at 280 nm. The mobile phases employed were a 0.01% aqueous solution of formic acid (A) and a 0.01% acetonitrile solution of formic acid (B). Gradient elution routines were 0–5 min, 9–12% B; 5–9 min, 12–20% B; 9–20 min, 20–46% B.

#### 4.9.2. Preparation of Standard Solution and Sample Solutions

For the preparation of analytical samples, 1.0 mg of chlorogenic acid, 4–O–Caffeoylquinic acid, hypericin, naringenin, and baicalin were accurately weighed and dissolved in 50% methanol to form a 1 mg/mL control solution; TZ samples of 21 batches were extracted from the TZ–TPC solution according to the method of preparation in ‘Section 4.2’ and evaporated and concentrated to obtain a dry paste, which was dissolved in 50% methanol to prepare analytical samples. All samples were filtered through a 0.22 µm microporous membrane.

#### 4.9.3. Fingerprinting and Methodological Validation

The TZ sample solution (TZ–1) was prepared according to the method under ‘Section 4.2’. The relative peak area (RPA) and relative retention time (RRT) of the common peaks were used as the detection indexes, and the RSD values were calculated for the methodological validation of the UHPLC fingerprints. Then, the sample solution (TZ–1) was repeatedly injected 6 times for precision analysis. Similarly, six samples of the same batch of sample solution (TZ–1) were prepared separately post-injection and evaluated for reproducibility. The same test solution was injected 0, 2, 4, 8, 12, and 24 h after sample preparation and tested under the chromatographic conditions under ‘Section 4.9.1’ to assess its stability. The RSD value of <3% [38] calculated from the methodological validation indicates that the analytical method is stable and reliable.

#### 4.9.4. Batch Fingerprint Analysis

Twenty-one batches of TZ-TPC samples were prepared according to the sample preparation method in ‘Section 4.2’, and the chromatograms were obtained under the chromatographic conditions in ‘Section 4.9.1’. The chromatograms of the 21 batches of TZ obtained from the UHPLC analysis system were imported into the “Chromatographic Fingerprint Similarity Evaluation System for Traditional Chinese Medicines” (2012 version). S1 was set as the control chromatogram with a time width of 0.5, which was generated according to the median, and the control chromatogram R and the common pattern superposition were obtained by multi-point correction and automatic matching for the calculation of chromatogram similarity. After obtaining the common peaks of each batch in the matching mode, the relative retention time and relative peak area were calculated, and the peak area data of chromatographic fingerprint profiles were Z–score standardised using SPSS 26.0 software and used for CA and PCA [27].

### 4.10. Spectrum–Effect Relationship

In the present study, the spectral relationship between the shared peaks of chromatographic fingerprints and the XOD inhibitory activity of TZ–TPC was investigated using GRA, OPLS–DA, and BCA. The common characteristic peak areas of 16 common peaks were designed as the comparison sequence using the XOD inhibition activity index as the reference sequence. Due to the inconsistency of dimensions between the values of the sequences to be tested, it may lead to unreliable evaluation results, which directly affects the correctness of the conclusions. Therefore, the raw data were pre-processed and normalised by Z–score using SPSS 26.0 software before the spectrum–effect relationship analysis of GRA, OPLS-DA, and BCA.

In the GRA, the reference sequences, comparison sequences, and absolute difference sequences were represented by {X_0_ (n)}, {X_i_ (n)}, and Δ_0i_(k) (i representing the number of samples), respectively. Designing the k as evaluation index sequence (k = 1, 2, …, n) to obtain the evaluation sequence {X_i k_} (i = 2; n = 19, in this study), the reference sequences and comparison sequences were recorded as {X_0_ (k)} and {X_i_ (k)} when the n = k (k representing peak), and the gray relational coefficients (η(k)) and gray relational grade(r) for each common peak were calculated at the same time according to the expression as follows [39]:

M = Δmax = maxmaxΔ_0i_(k) (1)

m = Δ min = min minΔ_0i_(k) (2)

Δ_0i_(k) = ∣X_0_(k) − X_i_(k)∣ (3)

η(k) = m + ρM/Δ_0i_(k) + *ρ*M (4)

r_i_ = r_i(0)_/[r_i(0)_ + ri_(k)_] (5)

where M and n are the optimal value and the worst value in the absolute difference sequence, and *ρ* is the resolution coefficient, which is usually chosen to be 0.5.

BCA was used to test whether the data were normally distributed using SPSS 26.0 software, Pearson’s parameters were selected for bivariate correlation test, and OPLS–DA was used to carry out orthogonal analyses of the relationship between the common peak data and the inhibition of XOD activity using SIMCA 14.1 software.

### 4.11. Molecular Docking

Molecular docking is a method for the identification of intermolecular interaction and prediction of the structure of receptor–ligand complexes by modelling the geometry of molecules and intermolecular forces [40,41]. The interaction between the screened active ingredient groups and XOD was further investigated using molecular docking software such as AutoDock Vina 4.2.6 (http://autodock.scripps.edu/ (accessed on 15 May 2024), PyMOL (https://pymol.org/ (accessed on 13 June 2024), PLIP (https://plip-tool.biotec.tu-dresden.de/plip-web/plip/index (accessed on 20 June 2024), and LigPlot^+^ (https://www.ebi.ac.uk/thornton-srv/software/LigPlus/ (accessed on 16 July 2024). The protein structure of XOD (PDB ID: 1FIQ) [29] was extracted from the Protein Data Bank (http://www.rcsb.org/ (accessed on 30 July 2024).

### 4.12. Statistical Analysis

In this study, the weighing of a sample of TZ and the determination of flavonoid content and antioxidant activity were repeated operations in parallel three times and averaged, and data were presented in the form of mean ± standard deviations (X ± SD). The collection, processing, and analysis of data were performed using the software SPSS 26.0 and Simca 14.1, and GraphPad 6.01 were applied to make figures.

## 5. Conclusions

In summary, in this study, the total polyphenol extraction method of TZ was optimised using a one-factor controlled variable method, and the optimal reaction conditions for XOD were investigated. The total polyphenol content of 21 batches of TZ and their IC_50_ values for the inhibition of XOD activity were also determined. In addition, the fingerprint of TZ was established by UHPLC, and sixteen common peaks were identified, of which five were recognised, and the similarity of each batch was greater than 0.9. In order to investigate the relationship between the components and the efficacy of the drug, mathematical correlation models, GRA, BCA, and OPLS–DA analyses were used to demonstrate that the inhibition of XOD by TZ is a synergistic effect of the components, and the more significant components were F2, F4, and F10. Molecular docking was used to fit the identified F2 and F10 components to the XOD proteins, and the results showed that the binding energies between the two were less than −7.5 kcal/mol, and the binding modes were diverse, including hydrogen bonding, hydrophobic forces, salt bridges, and so on. Meanwhile, the binding amino acid residue sites of the two are abundant, including arginine, serine, threonine, etc., indicating that the binding conformation of F2 and F10 small molecules to XOD protein is stable and has good affinity. It is speculated that small molecules may inhibit XOD activity through these amino acid residue sites. This study provides a reference for finding natural inhibitors of XOD and provides a theoretical basis for the development and utilisation of TZ medicinal materials.

## Figures and Tables

**Table 1:** One-factor examination of the extraction conditions of TZ–TPC (*n* = 3).

Order of Inspection	Variant	Prerequisite	Total Polyphenol Yield (mg/g)	Option
1	Withdrawal method	ultrasound extraction	4.348 ± 0.008	√
		reflux extraction	4.234 ± 0.013	
2	Extraction solvents	methanol	3.887 ± 0.018	
		ethanol	3.948 ± 0.008	√
3	Material–liquid ratio (g/mL)	1:30	2.673 ± 0.046	
		1:35	2.951 ± 0.026	
		1:40	3.199 ± 0.018	√
		1:45	2.343 ± 0.017	
		1:50	2.100 ± 0.010	
4	Ethanol volume fraction (%)	40	2.806 ± 0.10	
		50	3.060 ± 0.01	
		60	3.263 ± 0.06	√
		70	3.205 ± 0.01	
		80	3.118 ± 0.036	
5	Withdrawal time (min)	50	2.012 ± 0.046	
		60	2.136 ± 0.035	
		70	2.352 ± 0.035	
		80	2.468 ± 0.013	√
		90	2.174 ± 0.023	

Note: √ indicates that the condition is selected as optimal.

**Table 2:** Content of TZ–TPC in 21 different batches (*n* = 3).

NO.	TZ Batches	Total Polyphenol Yield (mg/g)	NO.	TZ Batches	Total Polyphenol Yield (mg/g)
1	TZ-14	7.454 ± 0.040	12	TZ-8	5.509 ± 0.023
2	TZ-11	7.245 ± 0.023	13	TZ-19	5.462 ± 0.061
3	TZ-17	6.790 ± 0.154	14	TZ-20	5.431 ± 0.074
4	TZ-6	6.689 ± 0.023	15	TZ-9	5.123 ± 0.027
5	TZ-13	6.635 ± 0.048	16	TZ-5	4.852 ± 0.088
6	TZ-4	6.597 ± 0.061	17	TZ-2	4.775 ± 0.053
7	TZ-12	6.381 ± 0.071	18	TZ-18	4.575 ± 0.081
8	TZ-10	6.373 ± 0.058	19	TZ-15	3.888 ± 0.000
9	TZ-3	6.249 ± 0.023	20	TZ-7	2.838 ± 0.013
10	TZ-16	5.979 ± 0.136	21	TZ-1	2.136 ± 0.013
11	TZ-21	5.624 ± 0.106			

**Table 3:** Screening of optimal conditions for the XOD reaction (*n* = 3).

Order of Inspection	Variant	Prerequisite	XOD Oxidation Rate (1 × 10^−4^)	Option
1	XOD concentration (U/L)	25	3.333 ± 1.155	
		50	4.333 ± 1.155	
		100	9.333 ± 1.155	
		200	11.667 ± 1.155	√
		400	3.667 ± 0.577	
2	Xanthine concentration (mg/L)	100	112.000 ± 5.292	
		120	175.667 ± 10.066	
		140	358.667 ± 3.215	
		160	412.667 ± 2.517	√
		180	372.333 ± 2.517	
		200	283.000 ± 1.000	
		220	229.000 ± 3.000	
3	Reaction temperature (°C)	15	165.000 ± 1.732	
		20	203.333 ± 8.083	
		25	264.333 ± 1.155	
		30	281.667 ± 7.024	√
		35	230.667 ± 9.292	
4	pH (PBS)	6	189.333 ± 2.517	
		6.5	218.667 ± 4.041	
		7	226.667 ± 5.859	
		7.5	229.667 ± 0.577	√
		8	217.333 ± 5.508	
		8.5	196.000 ± 3.606	

Note: √ indicates that the condition is selected as optimal.

**Table 4:** Ranking of IC_50_ values for inhibition of XOD activity by batch (*n* = 3).

Rank	Batch	1/IC_50_ (μg/mL)	Rank	Batch	1/IC_50_ (μg/mL)
Positive drug	Allopurinol	1.166 ± 0.059	11	TZ–20	0.582 ± 0.077
1	TZ–15	1.067 ± 0.091	12	TZ–13	0.579 ± 0.011
2	TZ–17	0.938 ± 0.201	13	TZ–14	0.576 ± 0.127
3	TZ–1	0.855 ± 0.052	14	TZ–7	0.548 ± 0.026
4	TZ–4	0.791 ± 0.027	15	TZ–12	0.494 ± 0.016
5	TZ–3	0.781 ± 0.126	16	TZ–16	0.454 ± 0.053
6	TZ–11	0.747 ± 0.116	17	TZ–10	0.449 ± 0.248
7	TZ–2	0.715 ± 0.407	18	TZ–21	0.427 ± 0.027
8	TZ–6	0.686 ± 0.183	19	TZ–8	0.422 ± 0.072
9	TZ–18	0.661 ± 0.059	20	TZ–19	0.412 ± 0.020
10	TZ–9	0.649 ± 0.022	21	TZ–5	0.381 ± 0.083

**Table 5:** GRA correlation ranking of TZ common peaks.

Rank	Peak	Relatedness Value	Rank	Peak	Relatedness Value
1	F14	2.571	9	F15	2.309
2	F7	2.475	10	F9	2.226
3	F3	2.473	11	F2	2.218
4	F10	2.464	12	F4	2.211
5	F5	2.376	13	F13	2.203
6	F1	2.349	14	F16	2.127
7	F8	2.338	15	F11	2.125
8	F6	2.323	16	F12	2.106

**Table 6:** Correlation coefficient values of two variables in BCA analysis and their significance analysis.

Peak	XOD		Peak	XOD	
	Pearson Correlation	Significance (Two–Tailed)		Pearson Correlation	Significance (Two–Tailed)
F1	−0.404	0.069	F9	−0.316	0.163
F2	0.081	0.727	F10	0.338	0.134
F3	−0.031	0.893	F11	0.270	0.236
F4	0.556 **	0.009 *	F12	−0.428	0.053
F5	−0.214	0.352	F13	−0.288	0.205
F6	0.032	0.890	F14	−0.280	0.219
F7	−0.043	0.853	F15	−0.281	0.217
F8	−0.463 *	0.034 *	F16	0.184	0.425

* Significant at the 0.05 level (two–tailed). ** Significant correlation at the 0.01 level (two–tailed).

**Table 7:** Information on binding sites for amino acid residues in small molecules and proteins for molecular docking.

Protein PDB Number	Ingredient	Type of Force	Amino Acid Residue	Residue Binding Site	Active Force
XOD-1FIQ	F2 4–O–Caffeoylquinic acid	Hydrophobic Interactions	TRP	336	3.93
			TRP	336	3.70
		Hydrogen Bonds	TRP	336	2.54
			LYS	422	3.23
			SER	425	2.65
		Salt Bridges	ARG	426	4.10
			LYS	433	4.8
			LYS	1228	5.45
		pi-Stacking	TRP	336	3.7
	F10 Naringenin	Hydrophobic Interactions	ILE	266	3.9
			THR	354	3.7
			ILE	358	3.6
		Hydrogen Bonds	GLU	45	2.8
			ASN	351	3.0
			ASN	351	4.0
			ARG	426	3.4
			SER	1225	4.1

**Table 8:** Spiking volume of different solutions.

NO.	PBS/mL	TZ Extracts/mL	XOD/mL	Thymine/mL
*A*_1_	0.390	0.010	0.050	0.350
*A*_2_	0.440	0.010	0.000	0.350
*A*_3_	0.400	0.000	0.050	0.350
*A*_4_	0.450	0.000	0.000	0.350

## Data Availability

The data presented in this study are available in the article and Supplementary Materials.

## Supplementary Materials

The following supporting information can be downloaded at: https://www.mdpi.com/article/10.3390/molecules29184288/s1, Figure S1: Plotting of gallic acid standard curve; Table S1: One-way analysis of total polyphenol content of 21 batches of TZ samples; Table S2: One-way ANOVA between IC_50_ values for XOD inhibition by TZ batches and allopurinol; Figure S2: Determination of the type of inhibition of XOD; Table S3: Methodological investigation of TZ fingerprinting; Figure S3: Methodological examination of fingerprint profiles. (A) Precision, (B) repeatability, and (C) stability; Table S4: Relative peak area of TPC; Table S5: Relative retention time of TZ total polyphenol fingerprints; Table S6: TZ 21 batch information; Table S7: Correlation coefficients for TZ BCA; Table S8: *p*-Value analysis for TZ BCA; Table S9: Correlation coefficients and ranking of VIP values in OPLS-DA; Figure S4: Venn diagram of correlation coefficients and VIP value screening components in OPLS-DA; Table S10: Molecular docking binding energy of two small molecules docked to XOD proteins; Table S11: Abbreviations.
