# Supplementary material for: Spectroscopic Relationship between XOD and TAOZHI Total Polyphenols Based on Chemometrics and Molecular Docking Techniques
Source: Molecules. 2024 Sep 10;29(18):4288. doi: 10.3390/molecules29184288 (PMC11433701; doi:10.3390/molecules29184288)
Supplement: Supplementary file 1 [file molecules-29-04288-s001.zip › molecules-3167689-supplementary.pdf]

Catalogues

Figure S1 Plotting of gallic acid standard curve. 2
Table S1 One-way analysis of total polyphenol content of 21 batches of TZ samples. 2
Table S2 One-way ANOVA between IC_{50} values for XOD inhibition by TZ batches and allopurinol. 7
Figure S2 Determination of the type of inhibition of XOD. 8
Table S3 Methodological investigation of TZ fingerprinting..8
Figure S3 Methodological examination of fingerprint profiles. (A) Precision (B) Repeatability (C) Stability. 9
Table S4 Relative peak area of TPC..9
Table S5 Relative retention time of TZ total polyphenol fingerprints. 10
Table S6 TZ 21 Batch Information 11
Table S7 Correlation coefficients for TZ BCA. 12
Table S8 p-value analysis for TZ BCA 13
Table S9 Correlation coefficients and ranking of VIP values in OPLS-DA. 13
Figure S4 Venn diagram of correlation coefficients and VIP value screening components in OPLS-DA...15
Table S10 Molecular docking binding energy of two small molecules docked to XOD proteins ..15
Table S11 Abbreviations 16

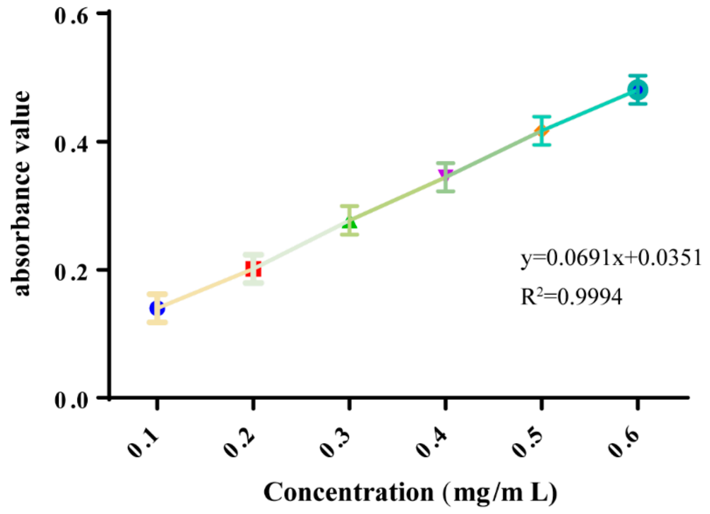

Figure S1 Plotting of gallic acid standard curve.

Table S1 One-way analysis of total polyphenol content of 21 batches of TZ samples.

Tukey's multiple comparisons test	Mean Diff.	95% CI of diff.	Significant?	Summary
TZ-1 vs. TZ-2	-2.64	-2.853 to -2.426	Yes	****
TZ-1 vs. TZ-3	-4.114	-4.327 to -3.900	Yes	****
TZ-1 vs. TZ-4	-4.461	-4.675 to -4.248	Yes	****
TZ-1 vs. TZ-5	-2.717	-2.930 to -2.503	Yes	****
TZ-1 vs. TZ-6	-4.554	-4.767 to -4.340	Yes	****
TZ-1 vs. TZ-7	-0.7024	-0.9159 to -0.4888	Yes	****
TZ-1 vs. TZ-8	-3.373	-3.586 to -3.159	Yes	****
TZ-1 vs. TZ-9	-2.987	-3.201 to -2.773	Yes	****
TZ-1 vs. TZ-10	-4.237	-4.451 to -4.024	Yes	****
TZ-1 vs. TZ-11	-5.11	-5.323 to -4.896	Yes	****
TZ-1 vs. TZ-12	-4.245	-4.459 to -4.031	Yes	****
TZ-1 vs. TZ-13	-4.5	-4.713 to -4.286	Yes	****
TZ-1 vs. TZ-14	-5.318	-5.531 to -5.104	Yes	****
TZ-1 vs. TZ-15	-1.752	-1.966 to -1.538	Yes	****
TZ-1 vs. TZ-16	-3.844	-4.057 to -3.630	Yes	****
TZ-1 vs. TZ-17	-4.654	-4.868 to -4.441	Yes	****
TZ-1 vs. TZ-18	-2.439	-2.653 to -2.225	Yes	****
TZ-1 vs. TZ-19	-3.327	-3.540 to -3.113	Yes	****
TZ-1 vs. TZ-20	-3.296	-3.509 to -3.082	Yes	****
TZ-1 vs. TZ-21	-3.489	-3.702 to -3.275	Yes	****
TZ-2 vs. TZ-3	-1.474	-1.688 to -1.261	Yes	****
TZ-2 vs. TZ-4	-1.822	-2.035 to -1.608	Yes	****
TZ-2 vs. TZ-5	-0.07718	-0.2908 to 0.1364	No	ns
TZ-2 vs. TZ-6	-1.914	-2.128 to -1.701	Yes	****
TZ-2 vs. TZ-7	1.937	1.724 to 2.151	Yes	****
TZ-2 vs. TZ-8	-0.7332	-0.9468 to -0.5197	Yes	****
TZ-2 vs. TZ-9	-0.3473	-0.5609 to -0.1338	Yes	****

TZ-2 vs. TZ-10	-1.598	-1.811 to -1.384	Yes	****
TZ-2 vs. TZ-11	-2.47	-2.683 to -2.256	Yes	****
TZ-2 vs. TZ-12	-1.605	-1.819 to -1.392	Yes	****
TZ-2 vs. TZ-13	-1.86	-2.074 to -1.647	Yes	****
TZ-2 vs. TZ-14	-2.678	-2.892 to -2.465	Yes	****
TZ-2 vs. TZ-15	0.8876	0.6740 to 1.101	Yes	****
TZ-2 vs. TZ-16	-1.204	-1.418 to -0.9905	Yes	****
TZ-2 vs. TZ-17	-2.014	-2.228 to -1.801	Yes	****
TZ-2 vs. TZ-18	0.2007	-0.01289 to 0.4142	No	ns
TZ-2 vs. TZ-19	-0.6869	-0.9005 to -0.4734	Yes	****
TZ-2 vs. TZ-20	-0.6561	-0.8696 to -0.4425	Yes	****
TZ-2 vs. TZ-21	-0.849	-1.063 to -0.6354	Yes	****
TZ-3 vs. TZ-4	-0.3473	-0.5609 to -0.1338	Yes	****
TZ-3 vs. TZ-5	1.397	1.183 to 1.611	Yes	****
TZ-3 vs. TZ-6	-0.4399	-0.6535 to -0.2264	Yes	****
TZ-3 vs. TZ-7	3.411	3.198 to 3.625	Yes	****
TZ-3 vs. TZ-8	0.741	0.5274 to 0.9545	Yes	****
TZ-3 vs. TZ-9	1.127	0.9133 to 1.340	Yes	****
TZ-3 vs. TZ-10	-0.1235	-0.3371 to 0.09008	No	ns
TZ-3 vs. TZ-11	-0.9957	-1.209 to -0.7821	Yes	****
TZ-3 vs. TZ-12	-0.1312	-0.3448 to 0.08236	No	ns
TZ-3 vs. TZ-13	-0.3859	-0.5995 to -0.1723	Yes	****
TZ-3 vs. TZ-14	-1.204	-1.418 to -0.9905	Yes	****
TZ-3 vs. TZ-15	2.362	2.148 to 2.575	Yes	****
TZ-3 vs. TZ-16	0.2701	0.05657 to 0.4837	Yes	**
TZ-3 vs. TZ-17	-0.5403	-0.7538 to -0.3267	Yes	****
TZ-3 vs. TZ-18	1.675	1.461 to 1.888	Yes	****
TZ-3 vs. TZ-19	0.7873	0.5737 to 1.001	Yes	****
TZ-3 vs. TZ-20	0.8181	0.6046 to 1.032	Yes	****
TZ-3 vs. TZ-21	0.6252	0.4116 to 0.8388	Yes	****
TZ-4 vs. TZ-5	1.744	1.531 to 1.958	Yes	****
TZ-4 vs. TZ-6	-0.09262	-0.3062 to 0.1210	No	ns
TZ-4 vs. TZ-7	3.759	3.545 to 3.972	Yes	****
TZ-4 vs. TZ-8	1.088	0.8747 to 1.302	Yes	****
TZ-4 vs. TZ-9	1.474	1.261 to 1.688	Yes	****
TZ-4 vs. TZ-10	0.2238	0.01026 to 0.4374	Yes	*
TZ-4 vs. TZ-11	-0.6483	-0.8619 to -0.4348	Yes	****
TZ-4 vs. TZ-12	0.2161	0.002542 to 0.4297	Yes	*
TZ-4 vs. TZ-13	-0.03859	-0.2522 to 0.1750	No	ns
TZ-4 vs. TZ-14	-0.8567	-1.070 to -0.6432	Yes	****
TZ-4 vs. TZ-15	2.709	2.496 to 2.923	Yes	****
TZ-4 vs. TZ-16	0.6175	0.4039 to 0.8310	Yes	****
TZ-4 vs. TZ-17	-0.193	-0.4065 to 0.02061	No	ns
TZ-4 vs. TZ-18	2.022	1.809 to 2.236	Yes	****

TZ-4 vs. TZ-19	1.135	0.9210 to 1.348	Yes	****
TZ-4 vs. TZ-20	1.165	0.9519 to 1.379	Yes	****
TZ-4 vs. TZ-21	0.9725	0.7589 to 1.186	Yes	****
TZ-5 vs. TZ-6	-1.837	-2.051 to -1.623	Yes	****
TZ-5 vs. TZ-7	2.014	1.801 to 2.228	Yes	****
TZ-5 vs. TZ-8	-0.6561	-0.8696 to -0.4425	Yes	****
TZ-5 vs. TZ-9	-0.2701	-0.4837 to -0.05657	Yes	**
TZ-5 vs. TZ-10	-1.521	-1.734 to -1.307	Yes	****
TZ-5 vs. TZ-11	-2.393	-2.606 to -2.179	Yes	****
TZ-5 vs. TZ-12	-1.528	-1.742 to -1.315	Yes	****
TZ-5 vs. TZ-13	-1.783	-1.996 to -1.569	Yes	****
TZ-5 vs. TZ-14	-2.601	-2.815 to -2.387	Yes	****
TZ-5 vs. TZ-15	0.9648	0.7512 to 1.178	Yes	****
TZ-5 vs. TZ-16	-1.127	-1.340 to -0.9133	Yes	****
TZ-5 vs. TZ-17	-1.937	-2.151 to -1.724	Yes	****
TZ-5 vs. TZ-18	0.2779	0.06429 to 0.4914	Yes	**
TZ-5 vs. TZ-19	-0.6097	-0.8233 to -0.3962	Yes	****
TZ-5 vs. TZ-20	-0.5789	-0.7924 to -0.3653	Yes	****
TZ-5 vs. TZ-21	-0.7718	-0.9854 to -0.5583	Yes	****
TZ-6 vs. TZ-7	3.851	3.638 to 4.065	Yes	****
TZ-6 vs. TZ-8	1.181	0.9673 to 1.394	Yes	****
TZ-6 vs. TZ-9	1.567	1.353 to 1.780	Yes	****
TZ-6 vs. TZ-10	0.3164	0.1029 to 0.5300	Yes	***
TZ-6 vs. TZ-11	-0.5557	-0.7693 to -0.3421	Yes	****
TZ-6 vs. TZ-12	0.3087	0.09516 to 0.5223	Yes	***
TZ-6 vs. TZ-13	0.05403	-0.1595 to 0.2676	No	ns
TZ-6 vs. TZ-14	-0.7641	-0.9777 to -0.5505	Yes	****
TZ-6 vs. TZ-15	2.802	2.588 to 3.015	Yes	****
TZ-6 vs. TZ-16	0.7101	0.4965 to 0.9237	Yes	****
TZ-6 vs. TZ-17	-0.1003	-0.3139 to 0.1132	No	ns
TZ-6 vs. TZ-18	2.115	1.901 to 2.328	Yes	****
TZ-6 vs. TZ-19	1.227	1.014 to 1.441	Yes	****
TZ-6 vs. TZ-20	1.258	1.045 to 1.472	Yes	****
TZ-6 vs. TZ-21	1.065	0.8516 to 1.279	Yes	****
TZ-7 vs. TZ-8	-2.671	-2.884 to -2.457	Yes	****
TZ-7 vs. TZ-9	-2.285	-2.498 to -2.071	Yes	****
TZ-7 vs. TZ-10	-3.535	-3.749 to -3.321	Yes	****
TZ-7 vs. TZ-11	-4.407	-4.621 to -4.194	Yes	****
TZ-7 vs. TZ-12	-3.543	-3.756 to -3.329	Yes	****
TZ-7 vs. TZ-13	-3.797	-4.011 to -3.584	Yes	****
TZ-7 vs. TZ-14	-4.616	-4.829 to -4.402	Yes	****
TZ-7 vs. TZ-15	-1.05	-1.263 to -0.8361	Yes	****
TZ-7 vs. TZ-16	-3.141	-3.355 to -2.928	Yes	****
TZ-7 vs. TZ-17	-3.952	-4.165 to -3.738	Yes	****

TZ-7 vs. TZ-18	-1.737	-1.950 to -1.523	Yes	****
TZ-7 vs. TZ-19	-2.624	-2.838 to -2.411	Yes	****
TZ-7 vs. TZ-20	-2.593	-2.807 to -2.380	Yes	****
TZ-7 vs. TZ-21	-2.786	-3.000 to -2.573	Yes	****
TZ-8 vs. TZ-9	0.3859	0.1723 to 0.5995	Yes	****
TZ-8 vs. TZ-10	-0.8644	-1.078 to -0.6509	Yes	****
TZ-8 vs. TZ-11	-1.737	-1.950 to -1.523	Yes	****
TZ-8 vs. TZ-12	-0.8722	-1.086 to -0.6586	Yes	****
TZ-8 vs. TZ-13	-1.127	-1.340 to -0.9133	Yes	****
TZ-8 vs. TZ-14	-1.945	-2.159 to -1.731	Yes	****
TZ-8 vs. TZ-15	1.621	1.407 to 1.834	Yes	****
TZ-8 vs. TZ-16	-0.4708	-0.6844 to -0.2572	Yes	****
TZ-8 vs. TZ-17	-1.281	-1.495 to -1.068	Yes	****
TZ-8 vs. TZ-18	0.9339	0.7203 to 1.147	Yes	****
TZ-8 vs. TZ-19	0.04631	-0.1673 to 0.2599	No	ns
TZ-8 vs. TZ-20	0.07718	-0.1364 to 0.2908	No	ns
TZ-8 vs. TZ-21	-0.1158	-0.3293 to 0.09779	No	ns
TZ-9 vs. TZ-10	-1.25	-1.464 to -1.037	Yes	****
TZ-9 vs. TZ-11	-2.123	-2.336 to -1.909	Yes	****
TZ-9 vs. TZ-12	-1.258	-1.472 to -1.045	Yes	****
TZ-9 vs. TZ-13	-1.513	-1.726 to -1.299	Yes	****
TZ-9 vs. TZ-14	-2.331	-2.544 to -2.117	Yes	****
TZ-9 vs. TZ-15	1.235	1.021 to 1.448	Yes	****
TZ-9 vs. TZ-16	-0.8567	-1.070 to -0.6432	Yes	****
TZ-9 vs. TZ-17	-1.667	-1.881 to -1.454	Yes	****
TZ-9 vs. TZ-18	0.548	0.3344 to 0.7616	Yes	****
TZ-9 vs. TZ-19	-0.3396	-0.5532 to -0.1260	Yes	****
TZ-9 vs. TZ-20	-0.3087	-0.5223 to -0.09516	Yes	***
TZ-9 vs. TZ-21	-0.5017	-0.7153 to -0.2881	Yes	****
TZ-10 vs. TZ-11	-0.8722	-1.086 to -0.6586	Yes	****
TZ-10 vs. TZ-12	-0.007718	-0.2213 to 0.2059	No	ns
TZ-10 vs. TZ-13	-0.2624	-0.4760 to -0.04885	Yes	**
TZ-10 vs. TZ-14	-1.081	-1.294 to -0.8670	Yes	****
TZ-10 vs. TZ-15	2.485	2.272 to 2.699	Yes	****
TZ-10 vs. TZ-16	0.3936	0.1801 to 0.6072	Yes	****
TZ-10 vs. TZ-17	-0.4168	-0.6304 to -0.2032	Yes	****
TZ-10 vs. TZ-18	1.798	1.585 to 2.012	Yes	****
TZ-10 vs. TZ-19	0.9108	0.6972 to 1.124	Yes	****
TZ-10 vs. TZ-20	0.9416	0.7281 to 1.155	Yes	****
TZ-10 vs. TZ-21	0.7487	0.5351 to 0.9622	Yes	****
TZ-11 vs. TZ-12	0.8644	0.6509 to 1.078	Yes	****
TZ-11 vs. TZ-13	0.6097	0.3962 to 0.8233	Yes	****
TZ-11 vs. TZ-14	-0.2084	-0.4220 to 0.005175	No	ns
TZ-11 vs. TZ-15	3.357	3.144 to 3.571	Yes	****

TZ-11 vs. TZ-16	1.266	1.052 to 1.479	Yes	****
TZ-11 vs. TZ-17	0.4554	0.2418 to 0.6689	Yes	****
TZ-11 vs. TZ-18	2.671	2.457 to 2.884	Yes	****
TZ-11 vs. TZ-19	1.783	1.569 to 1.996	Yes	****
TZ-11 vs. TZ-20	1.814	1.600 to 2.027	Yes	****
TZ-11 vs. TZ-21	1.621	1.407 to 1.834	Yes	****
TZ-12 vs. TZ-13	-0.2547	-0.4683 to -0.04113	Yes	**
TZ-12 vs. TZ-14	-1.073	-1.286 to -0.8593	Yes	****
TZ-12 vs. TZ-15	2.493	2.279 to 2.707	Yes	****
TZ-12 vs. TZ-16	0.4014	0.1878 to 0.6149	Yes	****
TZ-12 vs. TZ-17	-0.4091	-0.6226 to -0.1955	Yes	****
TZ-12 vs. TZ-18	1.806	1.593 to 2.020	Yes	****
TZ-12 vs. TZ-19	0.9185	0.7049 to 1.132	Yes	****
TZ-12 vs. TZ-20	0.9493	0.7358 to 1.163	Yes	****
TZ-12 vs. TZ-21	0.7564	0.5428 to 0.9700	Yes	****
TZ-13 vs. TZ-14	-0.8181	-1.032 to -0.6046	Yes	****
TZ-13 vs. TZ-15	2.748	2.534 to 2.961	Yes	****
TZ-13 vs. TZ-16	0.6561	0.4425 to 0.8696	Yes	****
TZ-13 vs. TZ-17	-0.1544	-0.3679 to 0.05920	No	ns
TZ-13 vs. TZ-18	2.061	1.847 to 2.274	Yes	****
TZ-13 vs. TZ-19	1.173	0.9596 to 1.387	Yes	****
TZ-13 vs. TZ-20	1.204	0.9905 to 1.418	Yes	****
TZ-13 vs. TZ-21	1.011	0.7975 to 1.225	Yes	****
TZ-14 vs. TZ-15	3.566	3.352 to 3.779	Yes	****
TZ-14 vs. TZ-16	1.474	1.261 to 1.688	Yes	****
TZ-14 vs. TZ-17	0.6638	0.4502 to 0.8773	Yes	****
TZ-14 vs. TZ-18	2.879	2.665 to 3.092	Yes	****
TZ-14 vs. TZ-19	1.991	1.778 to 2.205	Yes	****
TZ-14 vs. TZ-20	2.022	1.809 to 2.236	Yes	****
TZ-14 vs. TZ-21	1.829	1.616 to 2.043	Yes	****
TZ-15 vs. TZ-16	-2.092	-2.305 to -1.878	Yes	****
TZ-15 vs. TZ-17	-2.902	-3.116 to -2.689	Yes	****
TZ-15 vs. TZ-18	-0.6869	-0.9005 to -0.4734	Yes	****
TZ-15 vs. TZ-19	-1.575	-1.788 to -1.361	Yes	****
TZ-15 vs. TZ-20	-1.544	-1.757 to -1.330	Yes	****
TZ-15 vs. TZ-21	-1.737	-1.950 to -1.523	Yes	****
TZ-16 vs. TZ-17	-0.8104	-1.024 to -0.5969	Yes	****
TZ-16 vs. TZ-18	1.405	1.191 to 1.618	Yes	****
TZ-16 vs. TZ-19	0.5171	0.3036 to 0.7307	Yes	****
TZ-16 vs. TZ-20	0.548	0.3344 to 0.7616	Yes	****
TZ-16 vs. TZ-21	0.355	0.1415 to 0.5686	Yes	****
TZ-17 vs. TZ-18	2.215	2.002 to 2.429	Yes	****
TZ-17 vs. TZ-19	1.328	1.114 to 1.541	Yes	****
TZ-17 vs. TZ-20	1.358	1.145 to 1.572	Yes	****

TZ-17 vs. TZ-21	1.165	0.9519 to 1.379	Yes	****
TZ-18 vs. TZ-19	-0.8876	-1.101 to -0.6740	Yes	****
TZ-18 vs. TZ-20	-0.8567	-1.070 to -0.6432	Yes	****
TZ-18 vs. TZ-21	-1.05	-1.263 to -0.8361	Yes	****
TZ-19 vs. TZ-20	0.03087	-0.1827 to 0.2444	No	ns
TZ-19 vs. TZ-21	-0.1621	-0.3757 to 0.05149	No	ns
TZ-20 vs. TZ-21	-0.193	-0.4065 to 0.02061	No	ns

Table S2 One-way ANOVA between IC_{50} values for XOD inhibition by TZ batches and allopurinol.

Dunnett's multiple comparisons test	Mean Diff.	95% CI of diff.	Significant?	Summary
Allopurinol vs. TZ-1	0.3110	0.1414 to 0.4806	Yes	****
Allopurinol vs. TZ-2	0.3459	0.1763 to 0.5155	Yes	****
Allopurinol vs. TZ-3	0.3400	0.1704 to 0.5096	Yes	****
Allopurinol vs. TZ-4	0.3660	0.1964 to 0.5356	Yes	****
Allopurinol vs. TZ-5	0.7671	0.5975 to 0.9367	Yes	****
Allopurinol vs. TZ-6	0.4546	0.2850 to 0.6242	Yes	****
Allopurinol vs. TZ-7	0.6015	0.4319 to 0.7711	Yes	****
Allopurinol vs. TZ-8	0.7305	0.5609 to 0.9001	Yes	****
Allopurinol vs. TZ-9	0.5039	0.3343 to 0.6735	Yes	****
Allopurinol vs. TZ-10	0.6872	0.5176 to 0.8568	Yes	****
Allopurinol vs. TZ-11	0.4165	0.2469 to 0.5861	Yes	****
Allopurinol vs. TZ-12	0.6578	0.4882 to 0.8274	Yes	****
Allopurinol vs. TZ-13	0.5744	0.4048 to 0.7440	Yes	****
Allopurinol vs. TZ-14	0.5822	0.4126 to 0.7518	Yes	****
Allopurinol vs. TZ-15	0.1211	-0.04852 to 0.2907	No	ns
Allopurinol vs. TZ-16	0.7000	0.5304 to 0.8696	Yes	****
Allopurinol vs. TZ-17	0.1464	-0.02315 to 0.3160	No	ns
Allopurinol vs. TZ-18	0.4844	0.3148 to 0.6540	Yes	****
Allopurinol vs. TZ-19	0.7404	0.5708 to 0.9100	Yes	****
Allopurinol vs. TZ-20	0.5599	0.3903 to 0.7295	Yes	****
Allopurinol vs. TZ-21	0.7257	0.5561 to 0.8953	Yes	****

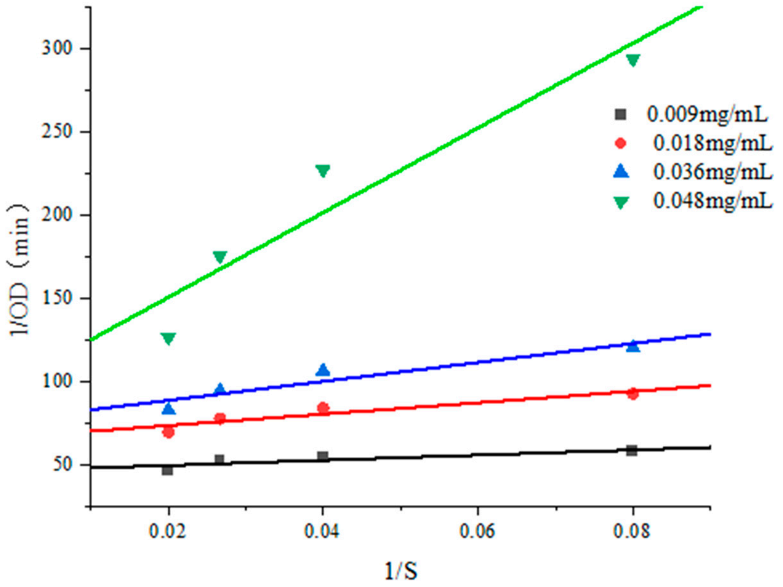

Figure S2 Determination of the type of inhibition of XOD.

Table S3 Methodological investigation of TZ fingerprinting**.**

Common peaks	Precision RSD (%)		Stability RSD (%)		Repeatability RSD (%)	
	Retention time	RPA	Retention time	RPA	Retention time	RPA
1	2.442	2.26	2.626	0.81	2.444	0.47
2	2.887	2.49	3.096	2.03	2.888	2.52
3	3.421	1.51	3.661	0.59	2.428	0.79
4	4.677	1.92	4.948	0.87	4.682	0.64
5	8.132	2.54	8.341	0.85	8.122	1.09
6	8.449	2.55	8.739	1.65	8.449	1.85
7	9.416	2.22	9.626	0.53	9.405	2.97
8	9.965	2.73	10.145	1.1	9.94	1.39
9	10.356	1.35	10.529	0.68	10.324	0.74
10	11.047	1.65	11.237	0.82	11.005	0.63
11	11.582	2.38	11.844	0.96	11.556	1.88
12	12.32	2.17	12.471	1.01	12.267	0.98
13	12.62	2.11	12.769	0.64	12.565	0.49
14	14.138	2.42	14.391	2.38	14.093	1.49
15	16.023	2.04	16.131	1.7	15.950	1.04
16	16.713	1.15	16.941	1.23	16.669	0.84

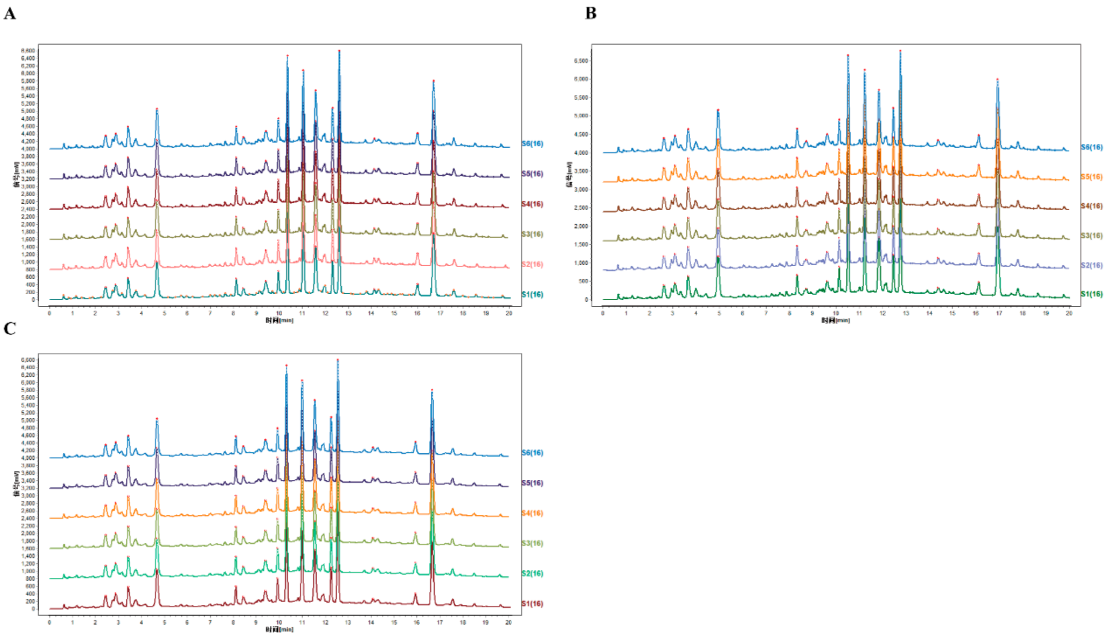

Figure S3 Methodological examination of fingerprint profiles. (A) Precision (B) Repeatability (C) Stability.

Table S4 Relative peak area of TPC

NO.	F1	F2	F3	F4	F5	F6	F7	F8(S)
S1	1.4659	12.2147	3.3223	9.8594	0.6830	1.5821	8.4129	1.0000
S2	1.2338	6.4397	3.1188	21.2192	0.8952	2.5838	4.9388	1.0000
S3	0.7929	0.6698	1.1179	2.3832	0.7963	0.3906	0.8042	1.0000
S4	0.4728	1.1281	0.8493	3.6501	1.2763	0.6350	0.9298	1.0000
S5	0.3669	3.9414	1.0247	4.5731	0.4759	1.1600	1.8811	1.0000
S6	0.8771	1.1898	1.3202	3.0176	0.9438	0.5288	0.9999	1.0000
S7	0.8032	8.2691	2.0305	9.1794	0.3561	0.9055	3.7178	1.0000
S8	1.7644	3.8525	2.1030	9.3090	1.4214	0.9557	2.6191	1.0000
S9	0.3031	0.3839	0.8401	0.9174	0.1723	0.2194	0.3694	1.0000
S10	3.2287	15.0666	6.3018	20.2922	1.1904	4.1660	3.1839	1.0000
S11	0.7364	2.0564	0.8341	2.7357	0.3039	0.6294	0.8921	1.0000
S12	0.8656	2.2686	1.0782	2.0497	0.4971	0.6989	0.2588	1.0000
S13	1.1002	2.2461	1.6072	4.6901	0.7092	0.5500	1.1549	1.0000
S14	0.3320	0.6387	0.2588	1.0615	0.4600	0.2504	0.3061	1.0000
S15	0.4064	3.6354	2.6891	1.5063	0.6141	0.2905	0.9731	1.0000
S16	0.4257	0.9170	1.5707	4.0497	0.3207	0.2754	0.6556	1.0000
S17	0.6722	0.7077	1.4461	2.3638	0.6721	0.2272	0.6950	1.0000
S18	0.4803	0.3161	0.6807	0.6231	0.3938	0.1625	0.3359	1.0000
S19	0.8357	0.5855	1.5142	4.3440	0.4442	0.2668	0.9509	1.0000
S20	0.4622	1.6417	1.3081	2.3888	0.1631	0.2449	0.2309	1.0000
S21	0.8805	1.2731	1.2994	3.2569	0.9279	0.2516	0.9308	1.0000
NO.	F9	F10	F11	F12	F13	F14	F15	F16
S1	3.7624	19.0038	9.8081	1.0162	2.0567	4.1664	1.0250	20.5611

S2	4.2494	22.1772	16.2463	1.2996	3.7064	1.0682	0.9522	23.4323
S3	2.8783	2.7073	2.2940	1.3938	3.4177	0.3222	0.6630	2.9768
S4	4.9560	3.6922	8.1146	0.8028	3.8721	0.5606	0.3798	9.1142
S5	3.2274	6.0585	8.1967	1.2228	2.8599	0.9206	0.4921	11.9141
S6	3.8098	3.5645	3.2049	1.6176	4.4815	0.3516	0.8101	4.6429
S7	3.2316	14.2701	14.0112	1.0409	2.3228	0.8951	0.2893	20.5694
S8	7.7860	9.7485	14.5387	1.3441	7.1733	1.2169	0.7761	18.9337
S9	1.7357	1.7757	2.1109	0.9306	1.3960	0.2007	0.5004	3.3659
S10	5.7220	19.2911	21.6027	1.2369	4.0930	1.6347	1.4432	31.7492
S11	2.8057	3.3958	5.3705	1.3596	2.8112	0.4080	0.4748	7.6526
S12	3.1802	2.6585	4.9886	1.3903	3.1971	0.4257	0.5448	7.5899
S13	3.1334	4.4593	5.0173	1.1105	2.7559	0.3947	0.4237	6.6225
S14	2.2881	1.2303	2.1670	1.2993	2.4431	0.1880	0.4176	2.7763
S15	3.8834	2.7612	2.1767	0.7440	2.1515	0.4154	0.5457	4.1858
S16	3.1296	5.1549	4.0049	0.7725	1.7294	0.2185	0.3273	6.0746
S17	3.3637	3.1878	2.8593	0.8711	2.3751	0.2543	0.4677	4.8216
S18	1.7687	0.9941	1.1739	0.8180	1.4514	0.1740	0.3216	2.0943
S19	2.5908	5.2100	7.6936	1.1213	2.0547	0.3879	0.5911	9.6486
S20	2.1401	3.0078	3.3272	0.9564	1.6799	0.3636	0.7807	5.4876
S21	3.0731	3.2319	3.7417	1.5171	3.5346	0.5123	0.7330	5.0006

Table S5 Relative retention time of TZ total polyphenol fingerprints.

NO.	F1	F2	F3	F4	F5	F6	F7	F8（S）
S1	0.2519	0.2870	0.3577	0.4838	0.8236	0.8538	0.9324	1.0000
S2	0.2563	0.2861	0.3559	0.4802	0.8199	0.8495	0.9308	1.0000
S3	0.2393	0.2821	0.3656	0.4917	0.8292	0.8567	0.9253	1.0000
S4	0.2506	0.2794	0.3475	0.4708	0.8093	0.8397	0.9412	1.0000
S5	0.2453	0.2742	0.3425	0.4635	0.8095	0.8290	0.9297	1.0000
S6	0.2501	0.2811	0.3499	0.4750	0.8183	0.8507	0.9459	1.0000
S7	0.2521	0.2820	0.3541	0.4782	0.8275	0.8488	0.9460	1.0000
S8	0.2516	0.2814	0.3513	0.4758	0.8105	0.8401	0.9354	1.0000
S9	0.2642	0.3010	0.3624	0.4877	0.8220	0.8589	0.9504	1.0000
S10	0.2550	0.2851	0.3547	0.4806	0.8201	0.8498	0.9465	1.0000
S11	0.2553	0.2862	0.3557	0.4825	0.8202	0.8499	0.9469	1.0000
S12	0.2514	0.2808	0.3465	0.4713	0.8101	0.8402	0.9409	1.0000
S13	0.2495	0.2799	0.3495	0.4733	0.8108	0.8410	0.9348	1.0000
S14	0.2442	0.2911	0.3734	0.4644	0.8075	0.8428	0.9337	1.0000
S15	0.2447	0.2801	0.3421	0.4659	0.8080	0.8387	0.9392	1.0000
S16	0.2470	0.2805	0.3457	0.4721	0.8164	0.8527	0.9442	1.0000
S17	0.2488	0.2948	0.3460	0.4717	0.8119	0.8459	0.9362	1.0000
S18	0.2472	0.2942	0.3462	0.4734	0.8180	0.8541	0.9454	1.0000
S19	0.2559	0.2905	0.3550	0.4809	0.8202	0.8580	0.9459	1.0000
S20	0.2507	0.2717	0.3475	0.4801	0.8239	0.8585	0.9595	1.0000
S21	0.2483	0.2696	0.3440	0.4496	0.8138	0.8376	0.9370	1.0000

NO.	F9	F10	F11	F12	F13	F14	F15	F16
S1	1.0377	1.1058	1.1646	1.2282	1.2569	1.4148	1.5935	1.6707
S2	1.0385	1.1079	1.1637	1.2318	1.2608	1.4035	1.5958	1.6704
S3	1.0435	1.1100	1.1577	1.2094	1.2391	1.3907	1.5716	1.6767
S4	1.0269	1.0955	1.1678	1.2187	1.2481	1.4019	1.5802	1.6839
S5	1.0177	1.0854	1.1577	1.2083	1.2370	1.3886	1.5669	1.6358
S6	1.0386	1.1076	1.1643	1.2321	1.2617	1.4193	1.5982	1.6747
S7	1.0387	1.1076	1.1629	1.2322	1.2610	1.4030	1.5952	1.6703
S8	1.0524	1.0943	1.1462	1.2167	1.2460	1.4011	1.5805	1.6522
S9	1.0380	1.1070	1.1624	1.2300	1.2589	1.3997	1.5734	1.6662
S10	1.0388	1.1076	1.1608	1.2314	1.2608	1.4020	1.5763	1.6677
S11	1.0384	1.1071	1.1626	1.2299	1.2595	1.4007	1.5740	1.6682
S12	1.0279	1.0961	1.1686	1.2199	1.2490	1.4023	1.5834	1.6541
S13	1.0271	1.0960	1.1492	1.2206	1.2508	1.4025	1.5814	1.6538
S14	1.0262	1.0945	1.1653	1.2199	1.2510	1.4001	1.5812	1.6499
S15	1.0271	1.0947	1.1675	1.2201	1.2490	1.4020	1.5841	1.6544
S16	1.0390	1.1065	1.1612	1.2329	1.2622	1.4163	1.5798	1.6710
S17	1.0266	1.0941	1.1654	1.2161	1.2447	1.3988	1.5783	1.6522
S18	1.0389	1.1081	1.1638	1.2346	1.2638	1.4182	1.5812	1.6748
S19	1.0389	1.1080	1.1602	1.2327	1.2619	1.4022	1.5769	1.6658
S20	1.0429	1.1160	1.1716	1.2340	1.2675	1.4087	1.6070	1.6728
S21	1.0275	1.1009	1.1473	1.2255	1.2454	1.3886	1.5768	1.6324

Table S6 TZ 21 Batch Information

No	Origin	Similarity	No	Origin	Similarity
TZ-1	Dangwu Town, Guiyang City, Guizhou Province 1	0.901	TZ-11	Jinsha County, Bijie City, Guizhou Province	0.991
TZ-2	Dangwu Town, Guiyang City, Guizhou Province 2	0.927	TZ-12	Dayegou, Bijie City, Guizhou Province	0.979
TZ-3	Dangwu Town, Guiyang City, Guizhou Province 3	0.933	TZ-13	Wangmo County, Xingyi City, Guizhou Province 3	0.993
TZ-4	Dangwu Town, Guiyang City, Guizhou Province 4	0.974	TZ-14	Guangshun Town, Changshun County, Guizhou Province	0.934
TZ-5	Dangwu Town, Guiyang City, Guizhou Province 5	0.980	TZ-15	Poxi Town, Jianhe County, Guizhou Province	0.903
TZ-6	Dangwu Town, Guiyang City, Guizhou Province 6	0.955	TZ-16	Nanshao Town, Jianhe County, Guizhou Province	0.978
TZ-7	Dangwu Town, Guiyang City, Guizhou Province 7	0.949	TZ-17	Taiyong Town, Jianhe County, Guizhou Province	0.982
TZ-8	Wangmo County, Xingyi City, Guizhou Province 1	0.991	TZ-18	Mindong Township, Jianhe County, Guizhou Province	0.926
TZ-9	Poxi Town, Jianhe County, Guizhou Province 1	0.977	TZ-19	Nanjia Town, Jianhe County, Guizhou Province	0.972

TZ-10	Wangmo County, Xingyi City, Guizhou Province 2	0.949	TZ-20	Nanming Town, Jianhe County, Guizhou Province	0.992
			TZ-21	Guanmo Town, Jianhe County, Guizhou Province	0.983

Table S7 Correlation coefficients for TZ BCA.

sample	F1	F2	F3	F4	F5	F6	F7	F8	F9
F1	1.000	-0.400	0.243	-0.266	0.582	-0.113	-0.297	0.502	0.449
F2	-0.400	1.000	0.185	0.073	-0.476	0.359	0.489	-0.501	-0.473
F3	0.243	0.185	1.000	-0.249	0.161	-0.342	-0.100	0.326	0.404
F4	-0.266	0.073	-0.249	1.000	-0.394	0.257	0.233	-0.705	-0.681
F5	0.582	-0.476	0.161	-0.394	1.000	-0.238	-0.219	0.495	0.795
F6	-0.113	0.359	-0.342	0.257	-0.238	1.000	0.067	-0.344	-0.407
F7	-0.297	0.489	-0.100	0.233	-0.219	0.067	1.000	-0.434	-0.475
F8	0.502	-0.501	0.326	-0.705	0.495	-0.344	-0.434	1.000	0.761
F9	0.449	-0.473	0.404	-0.681	0.795	-0.407	-0.475	0.761	1.000
F10	-0.506	0.341	-0.096	0.745	-0.650	0.069	0.635	-0.640	-0.768
F11	-0.417	-0.140	-0.631	0.300	-0.398	0.152	-0.236	-0.458	-0.442
F12	0.588	-0.538	0.081	-0.639	0.576	-0.212	-0.446	0.876	0.700
F13	0.605	-0.569	0.035	-0.557	0.844	-0.197	-0.445	0.654	0.830
F14	-0.165	0.503	-0.077	-0.147	-0.166	0.084	0.753	-0.225	-0.299
F15	0.525	-0.386	0.406	-0.561	0.436	-0.282	-0.408	0.782	0.612
F16	-0.541	0.200	-0.453	0.231	-0.758	0.202	-0.023	-0.486	-0.641
XOD	-0.404	0.081	-0.031	0.556	-0.214	0.032	-0.043	-0.463	-0.316

sample	F10	F11	F12	F13	F14	F15	F16	XOD
F1	-0.506	-0.417	0.588	0.605	-0.165	0.525	-0.541	-0.404
F2	0.341	-0.140	-0.538	-0.569	0.503	-0.386	0.200	0.081
F3	-0.096	-0.631	0.081	0.035	-0.077	0.406	-0.453	-0.031
F4	0.745	0.300	-0.639	-0.557	-0.147	-0.561	0.231	0.556
F5	-0.650	-0.398	0.576	0.844	-0.166	0.436	-0.758	-0.214
F6	0.069	0.152	-0.212	-0.197	0.084	-0.282	0.202	0.032
F7	0.635	-0.236	-0.446	-0.445	0.753	-0.408	-0.023	-0.043
F8	-0.640	-0.458	0.876	0.654	-0.225	0.782	-0.486	-0.463
F9	-0.768	-0.442	0.700	0.830	-0.299	0.612	-0.641	-0.316
F10	1.000	0.081	-0.708	-0.795	0.264	-0.557	0.321	0.338
F11	0.081	1.000	-0.417	-0.357	-0.244	-0.554	0.824	0.270
F12	-0.708	-0.417	1.000	0.847	-0.229	0.827	-0.571	-0.428
F13	-0.795	-0.357	0.847	1.000	-0.276	0.670	-0.682	-0.288
F14	0.264	-0.244	-0.229	-0.276	1.000	-0.095	0.042	-0.280
F15	-0.557	-0.554	0.827	0.670	-0.095	1.000	-0.578	-0.281
F16	0.321	0.824	-0.571	-0.682	0.042	-0.578	1.000	0.184
XOD	0.338	0.270	-0.428	-0.288	-0.280	-0.281	0.184	1.000

Table S8 p-value analysis for TZ BCA

sample	F1	F2	F3	F4	F5	F6	F7	F8	F9
F1	0.000	0.072	0.288	0.244	0.006	0.627	0.192	0.020	0.041
F2	0.072	0.000	0.421	0.753	0.029	0.110	0.024	0.021	0.030
F3	0.288	0.421	0.000	0.276	0.486	0.129	0.667	0.149	0.069
F4	0.244	0.753	0.276	0.000	0.077	0.261	0.309	0.000	0.001
F5	0.006	0.029	0.486	0.077	0.000	0.298	0.339	0.022	0.000
F6	0.627	0.110	0.129	0.261	0.298	0.000	0.774	0.127	0.067
F7	0.192	0.024	0.667	0.309	0.339	0.774	0.000	0.049	0.030
F8	0.020	0.021	0.149	0.000	0.022	0.127	0.049	0.000	0.000
F9	0.041	0.030	0.069	0.001	0.000	0.067	0.030	0.000	0.000
F10	0.019	0.130	0.679	0.000	0.001	0.767	0.002	0.002	0.000
F11	0.060	0.546	0.002	0.187	0.074	0.511	0.302	0.037	0.045
F12	0.005	0.012	0.727	0.002	0.006	0.356	0.043	0.000	0.000
F13	0.004	0.007	0.880	0.009	0.000	0.392	0.043	0.001	0.000
F14	0.475	0.020	0.739	0.526	0.473	0.716	0.000	0.326	0.188
F15	0.015	0.084	0.068	0.008	0.048	0.215	0.066	0.000	0.003
F16	0.011	0.384	0.039	0.314	0.000	0.379	0.922	0.025	0.002
XOD	0.069	0.727	0.893	0.009	0.352	0.890	0.853	0.034	0.163

sample	F10	F11	F12	F13	F14	F15	F16	XOD
F1	0.019	0.060	0.005	0.004	0.475	0.015	0.011	0.069
F2	0.130	0.546	0.012	0.007	0.020	0.084	0.384	0.727
F3	0.679	0.002	0.727	0.880	0.739	0.068	0.039	0.893
F4	0.000	0.187	0.002	0.009	0.526	0.008	0.314	0.009
F5	0.001	0.074	0.006	0.000	0.473	0.048	0.000	0.352
F6	0.767	0.511	0.356	0.392	0.716	0.215	0.379	0.890
F7	0.002	0.302	0.043	0.043	0.000	0.066	0.922	0.853
F8	0.002	0.037	0.000	0.001	0.326	0.000	0.025	0.034
F9	0.000	0.045	0.000	0.000	0.188	0.003	0.002	0.163
F10	0.000	0.728	0.000	0.000	0.247	0.009	0.157	0.134
F11	0.728	0.000	0.060	0.112	0.286	0.009	0.000	0.236
F12	0.000	0.060	0.000	0.000	0.317	0.000	0.007	0.053
F13	0.000	0.112	0.000	0.000	0.226	0.001	0.001	0.205
F14	0.247	0.286	0.317	0.226	0.000	0.682	0.856	0.219
F15	0.009	0.009	0.000	0.001	0.682	0.000	0.006	0.217
F16	0.157	0.000	0.007	0.001	0.856	0.006	0.000	0.425
XOD	0.134	0.236	0.053	0.205	0.219	0.217	0.425	0.000

Table S9 Correlation coefficients and ranking of VIP values in OPLS-DA.

Rank	Correlation coefficient		VIP value	
	Var ID (Primary)	M1. CoeffCS [1]	Var ID (Primary)	VIP
1	F2	0.3306	F13	1.5755
2	F7	0.2508	F9	1.5386

3	F10	0.2102	F10	1.4518
4	F6	0.1937	F2	1.3189
5	F14	0.1187	F4	1.0872
6	F4	0.0696	F16	1.0809
7	F16	-0.0004	F12	1.0704
8	F5	-0.0541	F8	1.0125
9	F12	-0.0548	F11	0.9373
10	F15	-0.0627	F7	0.8912
11	F8	-0.0674	F5	0.6869
12	F1	-0.0849	F15	0.5968
13	F3	-0.1339	F1	0.4929
14	F11	-0.1437	F14	0.4723
15	F13	-0.1857	F3	0.3312
16	F9	-0.2894	F6	0.2756

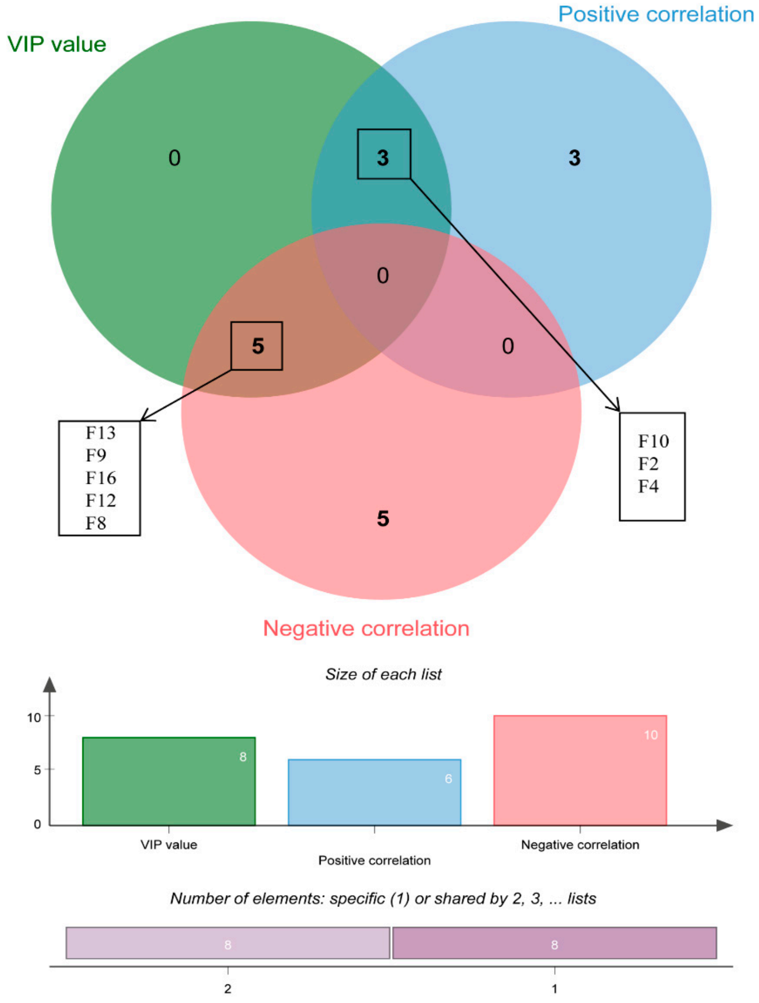

Figure S4 Venn diagram of correlation coefficients and VIP value screening components in OPLS-DA.

Table S10 Molecular docking binding energy of two small molecules docked to XOD proteins

mode	F2: 4-O-Caffeoylquinic acid			F10: Naringenin		
	affinity (kcal/mol)	dist from rmsd l.b.	best mode rmsd u.b.	affinity (kcal/mol)	dist from rmsd l.b.	best mode rmsd u.b.
1	-8.2	0	0	-8.7	0	0
2	-7.9	21.725	26.023	-8.5	3.878	8.462
3	-7.9	5.44	9.027	-8.3	3.368	6.862
4	-7.8	22.319	26.251	-8.3	16.873	20.145
5	-7.8	3.001	9.244	-8.2	3.81	8.726
6	-7.7	26.197	29.993	-8.1	5.042	7.086
7	-7.6	22.154	26.42	-8.0	24.746	26.722
8	-7.6	18.256	21.434	-7.9	20.395	20.987
9	-7.5	18.604	21.303	-7.8	39.688	41.979

Table S11 Abbreviations

TZ	TAOZHI
UHPLC	Ultra-High-Performance Liquid Chromatography
TPC	Total polyphenols
TZ-TPC	TZ total polyphenols
XOD	Xanthine oxidase
SECA	Spectral effect correlation analysis
RPA	Relative peak area
RRT	Relative retention time
PBS	Phosphate buffer saline
CA	Cluster analysis
PCA	principal component analysis
GRA	Grey Relation Analysis
BCA	Bivariate Correlation Analysis
OPLS-DA	Orthogonal Partial Least Squares Discriminant Analysis
VIP	Variable importance in projection
